# How to perform RT-qPCR accurately in plant species? A case study on flower colour gene expression in an azalea (*Rhododendron simsii* hybrids) mapping population

**DOI:** 10.1186/1471-2199-14-13

**Published:** 2013-06-24

**Authors:** Ellen De Keyser, Laurence Desmet, Erik Van Bockstaele, Jan De Riek

**Affiliations:** 1 Institute for Agricultural and Fisheries Research (ILVO)-Plant Sciences Unit, Caritasstraat 21, 9090, Melle, Belgium; 2 Department for Plant Production, Ghent University, Coupure links 653, 9000, Ghent, Belgium

**Keywords:** RT-qPCR, Flower colour, RNA quality, noRT, Standard curves, Reference genes, Gene expression, Pink

## Abstract

**Background:**

Flower colour variation is one of the most crucial selection criteria in the breeding of a flowering pot plant, as is also the case for azalea (*Rhododendron simsii* hybrids*).* Flavonoid biosynthesis was studied intensively in several species. In azalea, flower colour can be described by means of a 3-gene model. However, this model does not clarify pink-coloration. The last decade gene expression studies have been implemented widely for studying flower colour. However, the methods used were often only semi-quantitative or quantification was not done according to the MIQE-guidelines. We aimed to develop an accurate protocol for RT-qPCR and to validate the protocol to study flower colour in an azalea mapping population.

**Results:**

An accurate RT-qPCR protocol had to be established. RNA quality was evaluated in a combined approach by means of different techniques e.g. SPUD-assay and Experion-analysis. We demonstrated the importance of testing noRT-samples for all genes under study to detect contaminating DNA. In spite of the limited sequence information available, we prepared a set of 11 reference genes which was validated in flower petals; a combination of three reference genes was most optimal. Finally we also used plasmids for the construction of standard curves. This allowed us to calculate gene-specific PCR efficiencies for every gene to assure an accurate quantification. The validity of the protocol was demonstrated by means of the study of six genes of the flavonoid biosynthesis pathway. No correlations were found between flower colour and the individual expression profiles. However, the combination of early pathway genes (*CHS, F3H, F3'H* and *FLS*) is clearly related to co-pigmentation with flavonols. The late pathway genes *DFR* and *ANS* are to a minor extent involved in differentiating between coloured and white flowers. Concerning pink coloration, we could demonstrate that the lower intensity in this type of flowers is correlated to the expression of *F3'H*.

**Conclusions:**

Currently in plant research, validated and qualitative RT-qPCR protocols are still rare. The protocol in this study can be implemented on all plant species to assure accurate quantification of gene expression. We have been able to correlate flower colour to the combined regulation of structural genes, both in the early and late branch of the pathway. This allowed us to differentiate between flower colours in a broader genetic background as was done so far in flower colour studies. These data will now be used for eQTL mapping to comprehend even more the regulation of this pathway.

## Background

**Figure 1:**
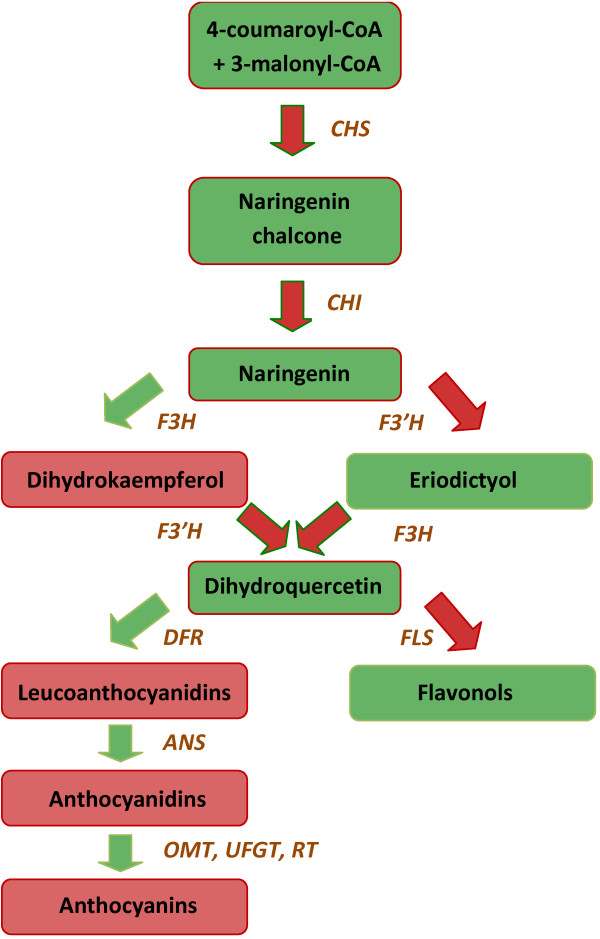
**Proposed flavonoid biosynthesis pathway in azalea.** The pathway only leads to the production of cyanidin pigments and is redrafted after [8,34]. *CHS: chalcone synthase; CHI: chalcone isomerase; F3H: flavanone 3-hydroxylase; F3'H: flavonoid 3'-hydroxylase; DFR: dihydroflavonol 4-reductase; ANS: anthocyanidin synthase; OMT:O-methyltransferase; UFGT: UDP-glucose:flavonoid 3-O-glucosyltransferase; RT: rhamnosyl transferase; FLS: flavonol synthase*.

As for all flowering plants, flower characteristics and especially flower colour are among the most important features for pot azalea (*Rhododendron simsii* hybrids) breeding. Flavonoids account for this pigmentation in azalea [1,2]. The flavonoid biosynthesis pathway is one of the best studied biochemical pathways in plants, especially in petunia and snapdragon [3-7]. Flavonoids are synthesized by a branched pathway that yields both coloured pigments (anthocyanins) and colourless co-pigments (flavonols). In De Cooman et al. [8], it was observed that the azalea co-pigment formation follows a slightly aberrant pathway compared to anthocyanin production (Figure 1). Anthocyanins tend to occur mainly as cyanidins, azaleatin is the most common flavonol in azalea [2]. Azalea flower colour ranges from purple through carmine red, red, pink and white. Furthermore, azalea flowers can also be picotee type, with a different-coloured centre and margin, or flecked. The latter is expected to be caused by transposon activities [9]. Flower colour segregation in azalea can be predicted by a Mendelian model encompassing 3 major genes (P, W & Q; [10]). Purple flower colour is dominant over all other colours and is encoded by P. In the absence of the allele for P, W differentiates between (red) coloured (W-) and white flowers (ww). Q encodes for co-pigmentation by means of flavonols; in combination with the allele for W it results in carmine red flowers. Red flowers are recessive for Q (qq). This model does not clarify the presence of pink flowers, but the authors suggested pink to be a gradation in pigment. Also Sasaki et al. [11] state that flower colour intensity is determined by the amount of anthocyanin present. By means of image analysis, De Keyser et al. [12] recently confirmed in azalea that pink can be seen as (carmine) red at a lower intensity level. Studying the gene expression levels of the flavonoid biosynthetic genes could be informative to shed a light on this pink mystery as well. By means of the transgenic approach, Nakamura et al. [13] created pink torenia plants by down regulation of *flavonoid 3′-hydroxylase* (*F3′H*) and *flavonoid 3′,5′-hydroxylase* (*F3′5′H*) genes and also Boase et al. [14] reported that the suppression of the latter gene resulted in reduced colour intensity. The past decade, genetic engineering is explored widely for the modification of floricultural plants (reviewed in [15]). Expression levels of the targeted genes were always determined in order to identify their correlation to the flower colour phenotype [13,16-18]. The exploration of natural flower colour differences by means of gene expression studies is only done between a limited number of genotypes, e.g. in cyclamen [19], *Ipomoea*[20], *Freesia hybrida*[21], azalea [22,23] or *Oncidium*[24]. No data are currently available on the consistent effect of the studied genes in other genotypes with the same flower colour. Moreover, the quantification methods used in the aforementioned studies are not the most accurate. Some studies still describe the use of Northern blots [18,24] or semi-quantitative RT-PCR (reversed transcription PCR) [16,19,21,23,24], others do use quantitative RT-PCR (RT-qPCR) but limit themselves to the comparative Cq (quantification cycle) method [25] in combination with the use of only a single non-validated reference gene. However, multiple, assay-validated reference genes are considered to be an essential component of a consistent RT-qPCR assay [26-30]. mRNA quantification can potentially be a very powerful and reliable technique for investigating gene expression, but only if handled thoughtfully [26,31]. Due to the sensitivity and in order to increase accuracy, the technique was optimised intensively the past decades at all crucial steps from RNA isolation up to the final quantification (reviewed in [31,32]). MIQE-guidelines (*M*inimum *I*nformation for Publication of *Q*uantitative Real-time PCR *E*xperiments; [26]) were set in order to stimulate the scientific community to quantify in an accurate manner and also to provide all essential data when publishing gene expression studies. However, in plant science, still too many papers on gene expression are published with inaccurate quantification [27-29,33], as was also illustrated for flower colour.

Hence, the aim of this paper is dual.

1. The establishment of a reliable RT-qPCR protocol for transcriptional profiling that can be applied in all plant species, even when only limited transcriptomic data are available. Optimisation at crucial steps is described into detail, with a focus on RNA quality, reference gene validation, the use of noRT (no Reversed Transcriptase) samples and the implementation of plasmid-derived standard curves for PCR efficiency correction.

2. Study of gene expression in relation to flower colour in an azalea mapping population to identify correlations that are not limited to specific genotypes but are consistent over the whole azalea gene pool. Ultimately, the idea is to use these gene expression data to study flower colour in a genetical genomics approach.

## Results

### Sampling

In azalea flowering, generally four developmental stages are considered: closed buds (stage 1), buds showing colour at the top but with the scales still present (stage 2), candle stage without any scales left (stage 3) and the opened flower (stage 4) [23]. Expression of both the early gene *CHS* (*chalcone synthase*) and the late gene *DFR* (*dihydroflavonol 4-reductase*) appeared to be highest in stage 3 [23], hence this stage was selected for the evaluation of flower colour gene expression. Nakatsuka et al. [34] report a higher expression in azalea for some of the early flavonoid biosynthesis genes in stage 2, but these are only 2-fold differences. We therefore preferred to quantify the expression profile of all genes on the same sample, which would allow us to correlate expression profiles of the different genes in our analysis.

### RNA quality control

Azalea RNA concentration varied tremendously between samples and was for some samples too low (Additional file 1) to test all genes in one RT-qPCR experiment. Hence we decided to extract RNA in duplicate from each sample. These technical replicates were then pooled after DNase treatment and purified together as one sample. RNA purity was measured spectrophotometrically. Contaminating proteins are displayed at an absorbance optimum of 280 nm, an A_260/280_ ratio above 1.8 is considered of an acceptable RNA purity although 2 would be optimal [35]. Concerning polysaccharide and polyphenol contamination, A_260/230_ is measured. A value of 2.5 means free of contamination [36], 2 is acceptable. However, the absorbance ratio’s only reflect RNA purity [26,37] but not RNA integrity [37]. Absorption ratios were satisfying, except for low-concentrated samples (<15 ng/μl) where both A_260/230_ and A_260/280_ were clearly decreased. The low absorption ratios could indicate the presence of potential inhibitors. However, the reliability of the measurement can also be questioned in case of low RNA concentrations.

**Figure 2:**
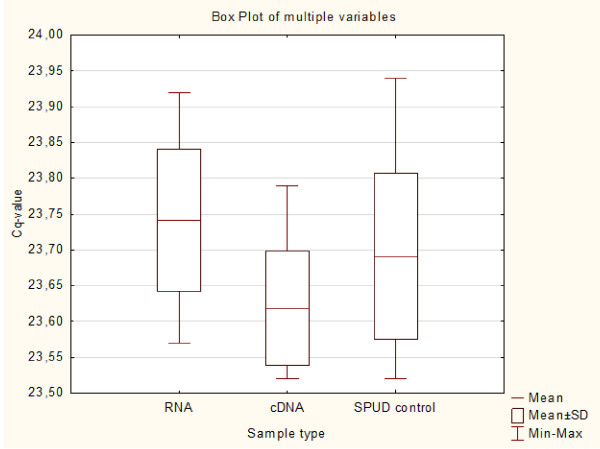
**Results of the SPUD assay.** Box plot of the Cq values obtained after the analysis of 3 sample types (RNA, cDNA and control) in a SPUD assay. For RNA/cDNA 14 different samples were measured in duplicate, 14 replicates were used for the SPUD control.

Performing a SPUD assay is considered to be the method of choice to evaluate the influence of inhibitory components on the RT-qPCR performance [32,38]. Therefore a subset of 14 randomly selected samples was used for a SPUD analysis. The difference in mean Cq-value between the SPUD control and RNA/cDNA samples did not exceed the variation within the SPUD control group (Figure 2) and remained below the proposed cut-off value of 1 Cq [39]. This confirmed that no PCR inhibitors were present in spite of the low absorption ratios in 3 samples (Additional file 1).

**Figure 3:**
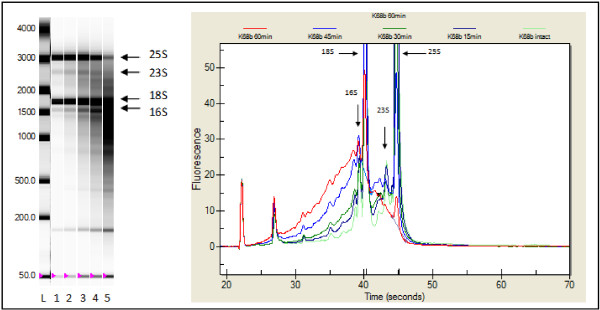
**RNA quality control with the Experion (Bio-Rad).** Electropherogram (right) and virtual gel-view (left) of an RNA degradation series that was constructed by heating an RNA sample for 0, 15, 30, 45 and 60 min at 80°C. The loading marker and small RNA band and cytoplasmic 18S and 25S as well as 16S and 23S chloroplast and mitochondrial ribosomal bands are indicated with arrows. Lanes: (L) size standard, (1) intact RNA, (2) 15 min, (3) 30 min, (4) 45 min, (5) 60 min. Intensity settings can vary between lanes.

Finally, RNA integrity was checked on the same subset of samples. In order to see how degradation evolved in our own material, we constructed a degradation series. A decrease of the ribosomal peaks and a shift in the electropherogram towards the so-called fast region [40] is clearly noticed (Figure 3). A visible degradation was also spotted on the gel-view (Figure 3). For low-concentrated samples, gel views were even the only reliable indicator for quality since the signal was too weak to verify on the electropherogram. Based on the degradation series, RNA was considered to be degraded when the 25S/18S rRNA ratio was below 1; degradation also becomes very well noticeable in the virtual gel view at this point (lane 4 and 5, Figure 3). According to these settings, all tested RNA samples were graded as good quality. Consequently, the robustness of our RNA isolation procedure from flower petals was demonstrated; RNA samples could even be placed for 15 hours at room temperature, without any visible degradation (data not shown). Hence, RNA quality results were extrapolated to all cDNA samples isolated from azalea flower buds in this study.

### Amplification specificity

Amplification of DNA in cDNA samples could result in an overestimation of the actual gene expression level of a gene or, even worse, in the false detection of expression. Developing primers spanning an intron or targeting exon-exon junctions can prevent co-amplification of DNA during RT-qPCR. Alignments with homologous sequences were made for all target genes (Table 1). No introns were present in *CHS*; intron-spanning primers were developed in *ANS* (*anthocyanidin synthase)* and *DFR*. In *FLS (flavonol synthase)* and *F3′H (flavonoid 3′-hydroxylase)* primers amplified a single exon but were located at the 3′ end of the sequence to reduce the influence of RNA degradation. The azalea *F3H* (*flavanone 3-hydroxylase)* fragment was too short and covered only a single exon. EST (Expressed Sequence Tags) sequences of the reference genes (Table 2) could not be evaluated for the presence of introns since their functional annotation was not specific enough. Hence, not all primers were intron-spanning and some introns were too small to prevent co-amplification of DNA [32]. Therefore DNA contamination had to be checked for after all. NoRTs were included for all samples and amplification was performed on these noRTs with all primer sets (both reference and target genes). In case of amplification of noRTs, contamination was considered to be negligible when the difference in Cq between the noRT and the sample was above seven cycles. In that case, at least 128-fold less contaminating DNA was present compared to cDNA. This is even above the five cycles that are the default setting for the same feature in qBase^+^ (Biogazelle), the software module that was developed by Hellemans et al. [41] for RT-qPCR data analysis. Only three samples amplified using the *DFR primers* and one sample using the *F3′H* primers were considered to be contaminated. Hence, these particular data were discarded from the dataset and only a single biological replicate was used instead for further calculations.

**Table 1:** Target genes

Code	**Gene**	**Acc. No.**	**Primer (5′-3′)**	**Ampl.**	**Position**
*ANS*	*anthocyanidin synthase*	AB289596	CCAAGAATCCGTCCGACTACA	65 bp	Exon1/2
			GGTTAGGCCTCTCAGGTGCTT		
*CHS*	*chalcone synthase*	AJ413277	TGGGAATCAACGGTTTTGGAA	151 bp	Exon1
			CTCGGGCTTAAGGCTCAACTT		
*DFR*	*dihydroflavonol 4-reductase*	AJ413278	CGTCATGAGGCTGCTTGAAC	151 bp	Exon1/2
			AAAGCTCCCTTCCTCGTTGAG		
*F3H*	*flavanone 3-hydroxylase*	AB289594	GGGCTCCAGGCCACTAGAG	87 bp	Exon2
			ATGGTCGCCCAAATTGACAA		
*F3′H*	*flavonoid 3′-hydroxylase*	AB289597	AAGAGCTGGACTCAATTGTTGGA	87 bp	Exon3
			CCTTGATGATGGCTTGGAGGTA		
*FLS*	*flavonol synthase*	AB289599	CAAGGATGTCATGGGCTGTGT	75 bp	Exon3
			CGTTAATGAGCTCCGGAATAGG		

Primer pairs for target genes were developed using Primer Express 2.0 (Applied Biosystems). EMBL accession numbers and the length of the amplicons are indicated. The position of the amplicons at the genomic DNA level is marked.

**Table 2:** Reference genes

**Gene**	**Acc. No.**	**Functional annotation**	**Primer (5′-3′)**	**Ampl.**
*GAPDH*	FN552706	*Glyceraldehyde 3-phosphate dehydrogenase*	TCGGAATCAACGGTTTTGGAA	151 bp
			CACTTGACCGTGAACACTGT	
HK5	AM932886	*Histone H3*	GAAACTCCCATTCCAGAGGCT	153 bp
			GCATGGATGGCACAGAGGTT	
HK47	AM932894	*Nucleosome assembly protein*	GGTATAGGATTGACAATCCCAAGG	151 bp
			CATTCAATCTCCGTCCCTATCG	
HK65	AM932901	*Protein kinase regulatory subunit γ*	CGGCAGTTAGGAGCTACCTCG	151 bp
			CCCTCACCGTCCACAACATAG	
HK92	FN552699	*Heterotrimeric G-protein,α subunit*	ATCACAGTCATCCATGCCAATG	151 bp
			CGCCGCCAATTTCTGATAGT	
HK96	AM932905	*Expansin*	AGGTTCACAATCAATGGCCAC	151 bp
			TGTTGCTCTGCCAATTCTGC	
HK112	FN552700	*3-deoxy-D-arabino-heptulo-sonate 7-phosphate synthase*	CTCCTCCCTTCCTCCCAATC	152 bp
			GTAACCGTTGTGCTCCCTACAGTC	
HK129	AM932909	*Protein phosphatase*	TGCAAAGATCGAATGCACGA	165 bp
			CCTGCAAACGGAACTCGAGA	
HK134	FN552701	*Chlorophyll a/b binding protein CP24 precursor*	CGGTTGCTCCCAAAAAGTCTT	158 bp
			CTCCGCTTCTCGGTACCACT	
HK156	FN552702	*Cytochrome P450 mRNA*	AGCCATGACCATCTTCGCTT	156 bp
			GGCGATGATGCAAACGAGTT	
HK164	FN552703	*Chlorophyll a/b binding protein*	AAAACCTCTTCTCTTGCAAACCAT	151 bp
			CTTGCCGACAGACTTCCTCAT	
HK173	FN552704	*Pyruvate dehydrogenase*	GGTGCGAGATTGGTATTTGGA	151 bp
			TTGAACTCCCAAAGCCATTGT	
HK190	FN552705	*Protein disulphide isomerase*	CGTATCGATCATCGGCTCGT	152 bp
			CACACCACGGAGCGTAGAACT	

Primer pairs for candidate reference genes were developed using Primer Express 2.0 (Applied Biosystems) based on EST-fragments (described by their EMBL accession number). The length of the amplicons and the putative function annotated to the sequences is indicated.

### Reference genes

The possible conservation of gene expression stability across different plant species [27] was an opportunity to select conventionally used reference genes in azalea. However, in a crop with only little sequence information available, this required degenerate PCR, with a low success-rate. Only *GAPDH* (*glyceraldehyde 3-phosphate dehydrogenase*) could be isolated as such. Hence, 13 fragments were selected based on putative functions from an azalea EST database [42] as candidate reference genes (Table 2). Amplification patterns of two of these genes (HK134 and HK190) did not satisfy in flower petals (data not shown). The expression of the 11 remaining reference genes was determined in petals of eight azalea cultivars and standard-curve derived quantities were imported into geNorm [30]. With a pair wise variation V_2/3_ of 0.145, the use of two reference genes seems sufficient (see Additional file 2). However, this value is nearby the proposed cut-off value of 0.15 and with V_3/4_ being only 0.108, three reference genes appeared to be most favourable for normalisation of gene expression in azalea flower buds. These validated reference genes have an optimal M-value (for homogeneous tissues) below 0.5 (M = 0.368 [41]) and belong to different functional classes. Hence they are not likely to be co-regulated, what enforces their trustworthiness for combination into a normalisation factor [30]. Unfortunately, when analysing the second assay, quite some noRTs amplified with one of the selected reference genes (HK173). Therefore this gene had to be eliminated as a reference gene for the final analysis. Hence, normalisation was done with a normalisation factor based on two reference genes (HK5 and HK129). The normalisation factor had a less optimal M-value of 0.524 over the three assays, still this solution was preferred over using unreliable expression data for normalisation.

### Standard curves

Plasmids containing the fragments of interest were used for the construction of a relative dilution series. Initially, reproducibility and stability of these dilution series was a major problem. However, this problem could be circumvented by linearization of the plasmids [43] and by diluting the linear fragments in a yeast tRNA solution. The addition of a carrier such as yeast tRNA prevents the loss of very little quantities in the smallest dilution steps [44]. In this way, the error on the linear regression of the dilution series was not worth mentioning. The SD(E) values (Additional file 3) were always below 0.01.

It is possible to analyze a standard curve only once for each gene and to apply the derived PCR efficiency in all further analysis. However, we preferred to work with run-specific amplification efficiencies to avoid the introduction of confounding technical variation. This was the best option, since amplification efficiencies of the individual standard curves clearly differ in time (Additional file 3), The PCR efficiency of e.g. HK129 varied between 0.94 and 0.81. The efficiencies for *F3′H* and certainly for *DFR* were far below the optimum, but by using the run-specific amplification efficiency, this difference in efficiency was accounted for and calculation errors were significantly reduced between assays.

### Flower colour gene expression

**Figure 4:**
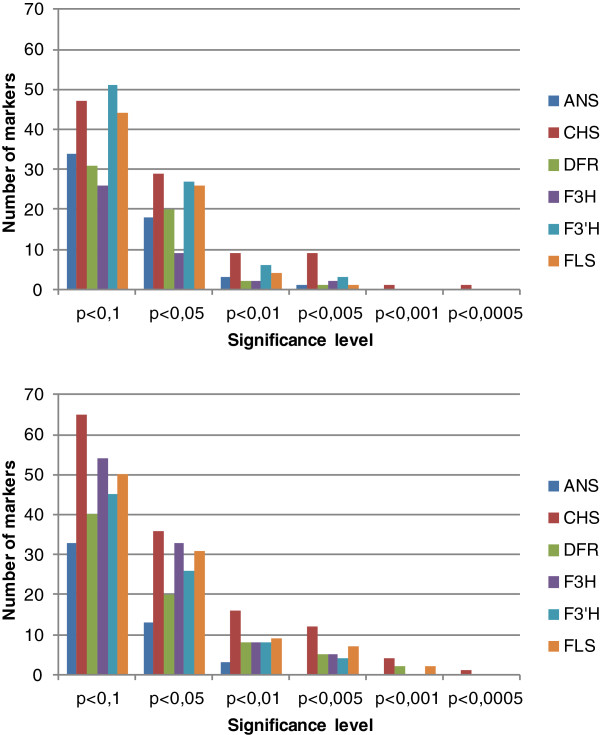
**Power analysis by means of Kruskal-Wallis eQTL mapping.** Preliminary eQTL mapping by means of Kruskal-Wallis analysis was performed in MapQTL^®^5 [91]. Two population sizes were compared: 49 siblings (upper panel) and 70 siblings (lower panel). For each gene (*ANS, CHS, DFR, F3H, F3′H* and *FLS*) the number of markers (vertical axis) that correlated at a certain significance level (horizontal axis) is given.

We aimed at finding gene expression differences for six key genes of the flavonoid biosynthesis pathway between four flower colour groups: white, red, carmine red and pink in an azalea mapping population. Initially we selected five seedlings from each flower colour group in combination with the (pink-coloured) parents of the crossing population (assay 1; see Additional file 1). No significant correlations were found between the colour grouping and the gene expression levels of the individual genes (data not shown). Since these data were in due course to be used for eQTL (expression Quantitative Trait Locus) mapping, we gradually expanded the dataset in order to determine the minimal sample size with sufficient power in eQTL mapping. First 29 samples were added to the dataset (assay 2; see Additional file 1) and Kruskal-Wallis analysis was performed to determine the power of eQTL mapping. This yielded only highly significant (p < 0.001) correlations for *CHS*. Eventually, we needed a total of 70 siblings to obtain enough power to detect (preliminary) eQTLs for 50% of the genes (Figure 4). We therefore considered 70 samples (+2 parents) to be sufficient for our gene expression study.

The results of all three assays were hence combined in a single dataset with 23 white flowers, 22 red, 19 carmine red and 8 pink ones. Due to the spread of the analysis over 3 different time points, inter-run calibration (IRC) was required to correct for potential run-to-run variation. Using (multiple) IRCs as advised by [41] was not feasible since these were not implemented consequently in every assay. Instead, the overall gene expression level per plate (and per gene) was used for inter-run calibration. The geometric mean was preferred over the arithmetic mean for calculating this IRC factor, as the former controls better for possible outlying values [30]. To verify whether our methodology did not introduce bias in the dataset, we decided to compare the outcome of both calculation methods. For this purpose, the samples of the total dataset were split up again after averaging the calibrated normalised relative quantities (CNRQ) of the biological replicates. All gene expression results, both CNRQ and NRQ (normalised relative quantities) per assay, are shown in Additional file 4. Mantel-analysis confirmed the consistency of the inter-run calibration method applied. The (C)NRQ values in both matrices were significantly correlated at the level of p = 0.001 for assay 2 and 3 and p = 0.004 for assay 1.

The mean difference in Cq values between technical replicates varied between 0.07 and 0.27 cycles. However, the variation in the technical replicates was considered negligible compared to biological variation. The fold differences of CNRQ values of some biological replicates varied noticeably (see Additional file 5). This was most pronounced for *F3′H* with a substantial higher mean and maximum fold difference. The latter is due to sample 234, which shows a lot of variation for the other genes as well. The biological variation in *DFR* expression is less pronounced, but with a mean/median of 1.76/1.38 still rather high.

No correlation could be found between the flower colour groups and gene expression levels (Table 3). Nevertheless, the expression of some genes appeared to be correlated to others, for *CHS* and *FLS* there was even a significant correlation with all other genes (Table 3). The flavonoid biosynthesis pathway can be partitioned among early and late pathway genes, but the breaking point differs between species [45,46]. In azalea, *F3H* and *F3′H* are considered as early pathway genes together with *CHS* and *FLS*; *ANS* and *DFR* are some of the late pathway genes [8]. Taking different combinations of early or late pathway genes as an input for discriminant analysis, some of these combinations appeared to be able to distinguish to a minor extent between flower colour groups (Table 4). Combining the expression of all 4 early pathway genes could classify 51.4% of the samples in the correct colour group. Co-pigmentation of flavonols cannot be visualised in white flowers and therefore the interpretation of the expression profiles in this group can be misleading, certainly for *FLS*. When white flowers were omitted from the dataset, already 65.3% of the samples could be assigned to the correct flower colour group based on the same combination of early pathway genes. In case we classified samples according to flower colour intensity (pink versus (carmine) red), the expression levels of the early pathway genes could assign over 85% of the samples correctly. Even the combination of all genes performed very well for this purpose. Interestingly, when we compared the *F3′H* gene expression levels between both groups (Mann–Whitney U-test), a significant difference (p = 0.0425) was found. When [13] down regulated this gene in torenia, flower colour turned to pink as well. These results confirm that *F3′H* gene expression is an important factor for the establishment of flower colour intensity in azalea as well.

**Table 3:** Spearman correlation analysis

	***ANS***	***CHS***	***DFR***	***F3H***	***F3′H***	***FLS***
**Colour**	0.123	−0.170	0.067	0.091	0.126	−0.152
***ANS***	1.000	0.329*	0.352*	0.549*	0.171	0.509*
***CHS***		1.000	0.309**	0.740*	0.617*	0.630*
***DFR***			1.000	0.078	0.214	0.307*
***F3H***				1.000	0.496*	0.582*
***F3′H***					1.000	0.418*

Non-parametric correlation was calculated between the log-transformed CNRQ values (geometric mean of biological replicates) of six genes and flower colour (white, pink, red and carmine red). *: significant at p < 0.01.

**Table 4:** Results of the assignments after discriminant analysis

**Genes included**	**Grouping variable**				
	**Colour**	**Colour (no white)**	**Intensity**	**W**	**Q**
***CHS/F3H/F3′H***	40.3%	59.7%	81.6%	59.7%	51.1%
***CHS/F3H/F3′H/FLS***	51.4%	65.3%	85.7%	58.3%	68.1%
***DFR/ANS***	27.8%	32.7%	57.1%	55.6%	51.1%
**All genes**	52.8%	55.1%	81.6%	55.6%	63.8%

Log-transformed CNRQ values of a combination of genes was used to calculate a discriminant function to predict classification according to 5 classes: colour (white, red, carmine red or pink); colour (no white; only red, carmine red and pink); intensity (pink versus (carmine) red); W (coloured versus white) and Q (co-pigmentation versus no co-pigmentation). The percentage of correctly assigned samples is presented.

When samples were classified according to their co-pigmentation pattern (Q/q [12]), again the combined information of the early pathway genes could discriminate best between both classes (68.1% correct classifications, Table 4). Also the combination of all six genes scores quite well in grouping the samples (63.8%). The difference between coloured and white flowers (W/w [12]) can be evaluated most reliable based on the expression of *CHS*, *F3H* and *F3′H*. The addition of *FLS* gene expression slightly reduces the information content (58.3% versus 59.7%), most likely due to the fact that flavonols have no impact on the phenotypic classification of W. However, when we look at the effect of the late pathway genes *ANS* and *DFR*, we can conclude that the expression of these genes is mainly involved in differentiating between white and coloured flowers as well.

## Discussion

### Optimisation of the RT-qPCR protocol

A good RT-qPCR experiment should always be based on a well-thought sampling protocol. Gene expression experiments essentially reflect a snapshot of RNA at the moment of extraction. Therefore, biological replicates are a prerequisite [26]. In this study, biological replicates were gathered on different flowers of a single plant. Indeed, sampling on two independent plants would have been a better approach since any influence of the physiological condition of the plant onto the overall gene expression would have been taken into account. However, when evaluating gene expression in a crossing population with only one plant per genotype, this is not an option. Growing all plants together at optimal conditions and sampling in a standardized way was therefore expected to be sufficient to fade out this effect as much as possible.

RT-qPCR has become the method of choice for gene expression analysis, but it suffers from considerable pitfalls, e.g. when it comes to evaluation of the RNA quality. Reporting on RNA quality assessment is one of the key-elements of the MIQE-guidelines [26] but is currently not done in 3 out of 4 published gene expression studies in plants [33]. Moreover, the results of the quality assessments are often not shown in the other 25%, although this information is crucial for the significance of the published results. Nevertheless, this parameter has a major impact on RT-qPCR performance [33,39,47,48], but there is no gold standard to define RNA quality and every method can have a different appreciation [39]. Absorption ratio’s only reflect RNA purity [37], whereas a SPUD-assay can evaluate for the inhibitory effect of these impurities [32,38]. Our results demonstrate that only looking at the absorption ratios can lead to wrong assumptions concerning the RNA quality. In spite of the low absorption ratios of several samples, no PCR inhibition was seen in the SPUD assay, indicating the acceptable quality of our samples. Assessing PCR efficiency in a test sample by serial dilution of the sample can be an alternative method to identify inhibition [32] but is not so obvious in case of low concentrated samples. D’haene and Hellemans [49] demonstrate that inhibitors can be derived from the shape of the amplification curve, but this is not an objective method. Hence, we advise to perform a SPUD assay on a representative subset of the samples every time a new sample type, treatment and/or extraction protocol is used.

To assess RNA integrity as well, microfluidic capillary electrophoresis was implemented. This technology recently gained interest in the plant RNA community (reviewed in [33]), but is partly based on the ribosomal peak ratio (28S/18S). Since the relationship between this ratio and mRNA integrity appears to be unclear [40,48,50,51], RIN (RNA Integrity Number [36]) and RQI (RNA Quality Indicator [52]) values that take into account the complete electropherogram were introduced as a more solid measure for RNA integrity. However, these values were initially assigned by using electropherograms of various mammalian tissues to train the software in an adaptive learning approach. In plants, no 28S rRNA is present, instead there is a 25S rRNA peak. In addition, total RNA in chloroplast-containing plant tissues also consists of 16S and 23S rRNA [53], adding 2 extra peaks. These rRNA peaks will be recognized as degradation peaks by the software, leading to a miscalculation of the RIN/RQI value and an underestimation of the true integrity of the material in plants. This is clearly seen in the result of Pico de Coana et al. [54]. Moreover, an optimal 28S/18S rRNA ratio of 2 is without any evidence extrapolated to plant 25S/18S rRNA [55]. These researchers rely on the software outputs, but they omit to look at the raw data to decide on the true quality of the RNA. Microfluidic capillary electrophoresis in plant science can be of great value (when the technology is available) but should always be restricted to a visual evaluation of the electropherograms and virtual gel views. The construction of a degradation series can then help to decide on the level of RNA integrity of specific samples.

Co-purification of traces of DNA during RNA extraction is inevitable, therefore noRT samples have been analysed in all cases. As is also asked for in the MIQE-guidelines [26], noRT results should always be given when gene expression data are published. However, far too often papers are published in which qPCR data are lacking results of the noRTs. How these authors (and the readers) can be sure that the so-called gene expression differences are not false positive signals? In the case the use of noRTs is described, it is not always clear what these noRTs exactly consist of. Some researchers just add RNA as a control in the RT-qPCR (e.g. [56,57]). However, to control in addition for DNA contamination during the cDNA synthesis step, we handled the RNA for noRT samples in exactly the same way as the normal samples. The same compounds were added, except off course the RT enzyme, as advised by Nolan et al. [32]. Suppliers of reversed transcriptase enzymes should provide special kits with additional buffers and primers for this purpose and this is unfortunately not always feasible. As an alternative, one could indeed use diluted RNA as a noRT sample and add the RT-reaction mixture as an additional sample in the analysis to control for potential contamination in this mixture. Even more crucial, in our opinion, is the analysis of noRTs with all primers. Often only a single gene is used to control for genomic DNA contamination [22,56-58]. The fact that in our dataset an individual sample was suffering from contamination when one specific gene was amplified, but not when the other genes were amplified, strengthens the need to test all primer sets on all noRT samples. Also Laurell et al. [59] state that the sensitivity towards genomic DNA contamination differs greatly between assays. These authors developed ValidPrime as an efficient alternative for the use of noRT controls, but currently no such assays are available for plant studies yet.

For normalisation of gene expression data, reference genes are indispensable [30]. The use of reference genes controls for variations in extraction yield, reverse-transcription and efficiency of amplification. It is without question that multiple, assay-validated reference genes are considered to be an essential component of a consistent qPCR assay [26], also in plant science [27-29]. In azalea, we aimed at developing a basic set of reference genes for application in all azalea gene expression studies. Czechowski et al. [60] demonstrated that the commonly used reference genes were not always the best candidates. Also GAPDH was not withdrawn as a reliable reference gene in our analysis. Therefore alternatives were looked for. Microarray data can be an ideal source of reference genes [61], but are lacking in azalea. Coker and Davies [62] took advantage of EST data for reference gene selection in tomato. Since a limited set of 62 ESTs was available in azalea [42], candidate reference genes were selected from this dataset. The proposed set of 11 azalea reference genes is a valuable toolbox for future qPCR research in azalea. However, each experimental condition demands a specific set of reference genes [63,64] and even different lab protocols seem to have an influence on reference gene selection [65]. Therefore, validation of this set in the desired tissues and conditions will be essential to select the appropriate assay-specific reference genes.

Several quantification strategies with altered normalisation methods are available, all depending on the PCR efficiency (E) for their calculations [25,41]. The quantification approach can have a serious impact on the final results [66]. Assuming an optimal PCR efficiency is not recommended [26,41]. The use of sample-specific amplification efficiencies [67-70] has become more common in RT-qPCR studies [71] since it allows quantification without standard curves. However, the outcome of using sample-specific amplification efficiencies can vary drastically depending on the settings and is reported to increase the random error [72]. Recently, Regier and Frey [66] demonstrated that using the average target specific efficiency (based on sample specific efficiency estimations) can be an alternative to the standard curve method in case a reliable algorithm is used (e.g. LinReg)**.** Nevertheless, the use of standard curves remains the most precise method [73,74]. Based on the equation of a standard curve, the qPCR efficiency can be calculated. In our study, plasmid DNA was used for standard curve construction. Hellemans et al. [41] advise to make the dilution series with a sample that mimics as much as possible the samples to be analysed in qPCR [41], most often this is a mixture of representative cDNA samples [57,75]. Plasmid DNA consists of a different sample matrix, what can result in altered efficiencies due to the presence of different kinds of inhibitory components [76]. However, the absence of PCR inhibitors was controlled for by means of the SPUD assay. Moreover, in absolute quantification studies the use of plasmid DNA to construct a dilution series is even preferred [77]. Especially in case of the limited availability of cDNA, plasmid DNA also has the advantage of being available plentiful and is therefore a valuable alternative for the construction of standard curves.

### Flower colour gene expression

Optimisation at all stages of the RT-qPCR has resulted in a reliable protocol for quantification of gene expression in azalea. We also aimed at studying the correlation between flower colour and the expression of candidate genes of the flavonoid biosynthesis pathway in a broader genetic background in contrast with what is currently reported in other ornamentals [19-22,24]. Moreover, we ultimately wanted to use flower colour as a model system for genetical genomics [78] in azalea. Most crucial was therefore the minimal required population size with sufficient power for eQTL mapping [79]. With 4 different flower colour groups, conventional power analysis [80] was not an option. But according to Shi et al. [81] even in small populations the power should already be sufficient to detect eQTLs. Therefore we started with a small subpopulation of 20 plants and gradually expanded to a final population of 70 siblings. This stepwise approach forced us to use an alternative method for inter-run calibration. The performance of a Mantel-test validated the approach for our assay. However, this method of inter-run calibration cannot automatically be considered to be trustworthy in other experiments. We believe that the rather small expression differences between our samples and genes had a significant impact here. Experiments in which large expression differences are measured are more likely to suffer from using the average gene expression as an inter-run calibrator and we therefore want to encourage the use of inter-run calibration as described in Hellemans et al. [41]. However, after validation with a Mantel-test, one could use the described methodology when lacking proper inter-run calibrators. The use of 3 biological replicates could have allowed to identify outlier values in some samples with high biological variation. However, these values do reflect the true variation present in the flower buds and can therefore not be neglected. These data clearly reinforce the substantial interest of using biological (rather than technical) replicates in every qPCR experiment.

The individual expression profiles were not discriminative enough to differentiate between colour groups. Also in other species, no such correlations have been reported since most studies limit themselves to the comparison of gene expression between few cultivars with different flower colours [19-22,24]. The use of multiple genotypes in each flower colour group certainly complicates the analysis. When the biological variation within a genotype is already substantial, detecting differences between genotypes is even harder. Only when the expression of *F3′H* was compared between pink and (carmine) red flowers, a significant expression difference was found. This implicates that there clearly is a link between the flower colour intensity and the *F3′H* expression. Similar conclusions can be drawn from the combined effect of early pathway genes (so including *F3′H*) on flower colour intensity, with very high percentages of correctly assigned genotypes. With a transgenic approach in torenia, Nakamura et al. [13] also demonstrated that the regulation of *F3′H* is crucial to manipulate flower colour intensity. Also *F3′5′H* is reported to be involved in pink [13,14] but this gene is only of interest for the production of dephinidin derivatives [82]. Delphidin pigments can be present in purple azalea flowers, but this colour was not present in the studied population. Therefore the expression of this gene was not determined. Besides these two flavonoid biosynthetic genes, pale-anthocyanin coloration can also be the result of a mutation in a putative *glutathione S-tranferase* gene that is responsible for the transport of pigments to the vacuole [83]. Therefore it would certainly be interesting to determine the expression of such transporter genes as well. HPLC measurements of the pigment types and concentrations could add even more to the elucidation of pink in azalea.

Also for the other genes, the combination of expression profiles was highly informative, since flower colour regulation is known to occur mainly via a coordinated transcriptional control of structural genes [5,7]). Especially the early pathway genes *CHS*, *F3H*, *F3′H* and *FLS* can discriminate rather well between the colour groups when white flowers are omitted from the analysis and these genes are most suited to differentiate for co-pigmentation as well. This makes sense, since the early pathway is indeed responsible for the production of the flavonols as co-pigments. To be able to include white flowers in the analysis, HPLC data would be needed to score for the presence of flavonols. The late pathway genes *ANS* and *DFR* are less informative but are still helpful for the classification of coloration. This could implicate that the difference between white and coloured flowers is situated rather at the regulation of the late pathway gene expression. Also in potato, *DFR* is known to be involved in the difference between white and coloured tubers [84] and Jung et al. [85] reported that the regulation of white pigmentation in potato is situated at the transcriptional level.

Due to the actual presence of gene expression differences that are related to the transcriptional regulation of the flavonoid biosynthetic pathway, these data are well-suited for eQTL mapping. For this purpose, not only the expression profiles of the individual genes but also the discriminant functions will be used as a first step towards *a priori* eQTL mapping [86] on the genetic map of the population under study [87]. As such, the gene expression information will be used in a genetical genomics approach [78] to evaluate the impact of the entire pathway on the flower colour. This can confirm the existence of a co-regulation network and will help to understand more the observed variation in flower colour. Moreover, the presence of markers for *myb*-functional genes on the genetic map can be valuable candidate genes potentially co-localising with flower colour eQTLs.

## Conclusions

To conclude, we are convinced that optimisation at crucial steps resulted in the development of a reliable protocol for gene expression analysis that is not only applicable to azalea, but can easily be used on other plant material as well. Currently in plant research, validated and qualitative RT-qPCR protocols are still rare. A pool of azalea reference genes was constructed, three of them are sufficient for normalisation of gene expression in flower petals, but the remaining genes can in the future also be used for normalisation in other azalea tissues, e.g. leaves and shoots. We also stressed on the importance of a multi-level RNA quality control, to evaluate both RNA purity and RNA integrity, with special attention for the bottlenecks for automated procedures on plant RNA. Furthermore, the co-amplification of contaminating DNA in few samples showed the importance of analysing noRT samples with all genes under study. Finally the advantages of using plasmid-derived standard curves in every analysis was demonstrated as well.

The accurate protocol resulted in the quantification of several flavonoid biosynthesis genes in a subset of 70 siblings of an azalea mapping population. The expression of *F3′H* could differentiate between pink and (carmine) red flower colour groups. The combined regulation of the early pathway genes clearly has an impact on the co-pigmentation and the late pathway genes *ANS* and *DFR* are to a minor extent involved in differentiating between white and coloured flower phenotypes. These gene expression profiles will now be used as eQTLs to study flower colour in a genetical genomics approach. This might help us to point-out the actual genes that are encompassed in W and Q. Providing more detailed data on pigment composition (HPLC) in the petals of the different genotypes could even add an additional level (mQTLs or metabolite QTLs) of information to this map-based approach.

## Methods

### RNA isolation

RNA was isolated from flower buds in the candle stage (25–30 mm) [23] of 70 siblings of the ‘GxH’ crossing population [87] and both parents (‘98-13-4’ and ‘Sima’). From each plant, two individual buds were sampled (a and b) as biological replicates. For reference gene selection, candle stage flower buds of eight azalea cultivars (‘Hellmut Vogel’ and seven of its flower colour sports: ‘Paloma’, ‘Hector’, ‘Mw. Troch’, ‘Nordlicht’, ‘Terra Nova’, ‘Zalm Vogel’ and ‘Super Nova’) displaying a range of colours were used. Approximately 70 mg of petal tissue (other bud organs were carefully removed) was weighed per sample in duplicate in pre-cooled 2 ml safe-lock tubes (Eppendorf). Three zirconium beads were added to the tubes and the plant material was crushed in a pre-cooled block of the Retsch Tissuelyser (Qiagen) for 2 times 30 s at 30 Hz. After a short centrifugation (30 s, 4°C, full speed), the tubes were placed on ice and RNA was isolated according to the protocol of the RNAqueous kit® (Ambion) in combination with the Plant RNA Isolation Aid (Ambion). Elution was done in three steps (40/25/25 μl) and eluents were pooled. DNase treatment occurred on 80 μl of RNA with the DNA-*free* kit (Ambion). 10 μl DNaseI buffer and 1.5 μl rDNaseI were added, followed by an incubation step of 30 min at 37°C. DNase Inactivation Reagent (10 μl) was added and samples were incubated for 2 min at room temperature. After centrifugation (90 s, 10000 g) the supernatant was transferred to a new tube. Duplicate samples were finally pooled and purified [88] using 0.3 M Sodium Acetate pH5.5 (Ambion). Two and a half volumes of 100% EtOH was added and samples were incubated for at least 15 min at −80°C or overnight at −20°C. Supernatant was removed after 25 min centrifugation (14000 rpm, 4°C) and 1 ml 70% EtOH was added. Again tubes were centrifuged for 20 min at the same conditions and supernatant was discarded. The RNA pellet was dried in a vacuum-desiccator and resolved in 25 μl of RNase-free water. Samples were stored at −80°C until cDNA synthesis.

### RNA quantity/quality

RNA was quantified by means of the NanoDrop spectrophotometer (Isogen). The presence of inhibitory components was evaluated (on a subset of 14 samples, Additional file 1) by means of the SPUD-assay developed by [30,32]. A stock solution of 5 μM of the 101 bp SPUD amplicon (Sigma) was diluted 1/10^8^ in yeast tRNA (50 ng/μl; Invitrogen). 0.5 μl of the diluted amplicon, 0.48 μM of both forward and reverse SPUD primers (Invitrogen), 0.1 μM of the dual-labelled (Fam-Tamra) SPUD probe (MWG-Biotech) and 1× LightCycler480 Probes Master Mix (Roche) was combined in a total volume of 10 μl in a white 384-well plate (Roche). For each sample, 1 μl of RNA or 2 μl of cDNA was added and all samples were analysed in duplicate. In the SPUD control samples, no RNA or cDNA was added; NTCs (No Template Control) were included as well. Plates were sealed with an adhesive film. Cycling conditions in the LightCycler480 (Roche) were 10 min at 95°C, followed by 45 cycles of 10s 95°C, 30 s 60°C and 1s 72°C. Fluorescence data were recorded every cycle at the end of the annealing/elongation step at 60°C. Data were analysed using the LightCycler480 software version 1.5 (Roche). Cq-values were exported to Microsoft Excel for further calculations. Finally, RNA quality and quantity was also determined on the same subset of samples using the Experion microfluidic capillary electrophoresis system (Bio-Rad) in combination with the RNA StdSens Chips (Bio-Rad). A degradation series was prepared by heating an RNA sample for 15, 30, 45 and 60 min at 80°C in a PCR machine.

### Reverse transcription

First strand cDNA synthesis was performed with the SuperScript III First-Strand Synthesis SuperMix (Invitrogen) according to the manufacturers protocol and starting from 100 ng of RNA or 6 μl for low-concentrated samples (< 17 ng/μl). Oligo(dT)_20_ was used for priming and all incubations occurred in a Perkin Elmer 2720 (Applied Biosystems). As a control for DNA contamination, noRTs were created in the same way as samples, except for the SuperScript III/RnaseOUT Enzyme Mix that was omitted in these cases. Both cDNA and noRT samples were diluted 1/3 and stored at −20°C.

### Reference genes

Homolog’s of commonly used reference genes (*ubiquitin, GAPDH, β-actin, α-6-tubulin, TATA-box binding protein, elongation factor α*) were searched for in azalea with degenerate primers; gene-isolation was only doing well for *GAPDH*. The fragment was cloned using the TOPO TA Cloning Kit (Invitrogen) and sequenced in order to develop specific RT-qPCR primers (Table 2). Twelve candidate reference genes were selected out of 62 annotated genes from a *Rhododendron simsii* hybrid ‘Flamenco’ EST library [42] and qPCR primers were developed with melting temperatures 58-60°C, primer lengths 20–24 bp and amplicon lengths 151–165 bp. (Primer Express 2.0, Table 2). Primers were at first tested on the EST containing plasmids. Primer pairs that amplified the proper fragment were, together with *GAPDH* primers, tested in duplo in a RT-qPCR assay on cDNA from flower petals of 8 azalea cultivars. PCR analysis was carried out in an ABI7000 thermocycler (Applied Biosystems). Amplification mixture consisted of 12.5 μl of SYBR Green I Master Mix (Applied Biosystems), 7.5 pmol of both primers and 2 μl cDNA in a total volume of 25 μl. Cycling conditions were 2 min 50°C, 10 min 95°C and 40 cycles of 15 s 95°C and 1 min 60°C. For melting curve analysis, cycling conditions were 15 s 95°C, 15 s 60°C followed by ramping from 60°C to 95°C with a ramp speed of 2% and a final step of 15 s 95°C. Cq-values were averaged and transformed to quantities using standard curves. These data were used for reference gene selection using geNorm software [30].

### Standard curves

Amplified fragments of both reference and target genes were cloned using the TOPO TA Cloning Kit (Invitrogen) containing TOP10F’ chemically competent cells and the pCR2.1-TOPO cloning vector. For *CHS* and *DFR*, full length cDNA sequences were previously cloned [23]. Plasmid DNA was purified (GFX *Micro* Plasmid Prep Kit, Amersham) and linearised using 10 U of *Hind*III (Invitrogen) for 2 h at 37°C, followed by an enzyme inactivation step for 10 min at 70°C. The stock concentration of plasmids was diluted to a working solution of 1 ng/μl in 50 ng/μl yeast tRNA (Invitrogen). Standard curves were constructed as six log10 dilutions of this working solution in yeast tRNA (50 ng/μl). To prevent extrapolation, the range of the standard curve was set to cover Cq values of the cDNA samples. It must also be strengthened that the diluted aliquots were never stored longer as 24 h at 4°C to preserve quality [89] were and prepared newly from the same stock of plasmid DNA stored at −20°C if needed again later. Standard curves were used for calculation of PCR efficiencies (E = 10^(−1/slope)^ -1).

### Quantification

Six RT-qPCR primer sets were developed in azalea for genes coding for key enzymes in the flavonoid biosynthesis pathway: *chalcone synthase* (*CHS)*, *flavanone 3-hydroxylase* (*F3H)*, *flavonoid 3′-hydroxylase* (*F3′H*), *anthocyanidin synthase* (*ANS)*, *dihydroflavonol 4-reductase* (*DFR)* and *flavonol synthase* (*FLS)* (Table 1)*. CHS* and *DFR* were *R. simsii* hybrid sequences [9], the others from *R. Xpulchrum*[34]. Primers were designed using Primer Express 2.0 (Applied Biosystems). Primers were targeted to the 3′ end and preferably spanning an intron. Intron/exon positions were predicted based on homologies with poplar or *Arabidopsis* sequences. Small amplicon sizes were preferred because this gives more consistent results [48]. All samples, noRTs, NTCs and standard curves were measured in duplicate in a LightCycler480 (Roche). In a white 384-well plate (Roche), 375 nM of each primer and 5 μl of LightCycler480 SYBR Green I Master (Roche) was used with 2 μl of sample in a total volume of 10 μl. Plates were sealed with an adhesive film. Cycling conditions were 5 min at 95°C, followed by 40 cycles of 10 s 95°C, 12 s 60°C and 10 s 72°C. Data acquisition was done at the end of every cycle. Melting curve analysis was performed as follows: 5 s 95°C, 1 min 65°C and heating to 97°C with a ramp rate of 0.06°C/s. Data acquisition occurred 10 times for every °C. Data were analysed using the LightCycler480 software version 1.5 (Roche). We started with gene expression analysis on 20 siblings and both parent plants. In a second phase, 29 new siblings were analysed and finally a third assay was run with 21 seedlings for gene expression analysis (See Additional file 1). Within an assay, the sample-maximisation method was preferred and samples were analysed in a single plate per gene. The 2^nd^ derivative method of Luu-The et al. [90] was selected for Cq determination in every run. Cq-values were exported to Microsoft Excel; technical replicates were averaged geometrically. For combining the 3 assays, the overall gene expression level per plate and per gene (geometric mean) was used for inter-run calibration. Gene specific amplification efficiencies derived from standard curves and a normalisation factor [30] based on two validated reference genes (HK5 and HK129) was used for calculation of (calibrated) normalised relative quantities ((C)NRQ). Biological replicates were averaged geometrically as well.

### Data analysis

Log-transformed data were used as an input for statistics. SPSS Statistics 19 software package was used for all statistical data analysis. Kruskal-Wallis (in MapQTL®5 [91]) was used as an alternative for power analysis to determine the required population size. Power was sufficient when at least half of the genes correlated with markers at the level of p < 0.001. To verify the inter-run calibration method, two calculation methods were compared for each assay: standard quantification in the individual assay (NRQ-values) and the same subset of samples calculated within the global dataset of 72 samples (CNRQ-values). Bivariate spearman correlation coefficients were calculated between log-transformed values of all samples for every gene, resulting in assay-specific correlation matrices. Correlation matrices of comparable datasets were used as an input for Mantel analysis [92] by means of the Mantel nonparametric test calculator [93].

## Competing interests

The authors declare that they have no competing interests.

## Authors’ contributions

EDK was responsible for design of the study, data analysis and statistics and drafted the manuscript; LD was involved in the acquisition of data and assisted in data analysis; EVB participated in the study’s design and contributed to editing the manuscript; JDR conceived the study, participated in data analysis and helped to draft the manuscript. All authors read and approved the final manuscript.

## Supplementary Material

Additional file 1 **RNA concentration and purity.** Description: RNA quantity and purity was measured of each biological replicate per sample using a NanoDrop spectrophotometer. For each sample, the assay is indicated in which the sample was analysed. Flower colour is indicated as well (0 = white, 1 = red, 2 = carmine red, 3 = pink). Samples used for analysis in the SPUD-assay and the Experion are indicated with an *. Click here for file

Additional file 2 **Evaluation of the optimal number of reference genes for normalization.** Description: A cut-off value of 0.15 is proposed (top panel). Average expression stability (M) of the reference genes tested in azalea. M is calculated at each step during stepwise exclusion of the least stable reference gene. Genes are ranked from the least (left) to the most stable (right). Only genes with an M-value < 0.5 are valid in homogeneous samples (lower panel). Both graphs are generated in GeNorm [30]. Click here for file

Additional file 3 **PCR efficiencies of the standard curves.** Description: Summary of slopes and derived PCR efficiencies (E) of the standard curves of dilution series analysed on different plates in 3 independent assays. E and the standard deviation on E (SD(E)) were calculated according to the formulas described in Hellemans et al. [41]. Click here for file

Additional file 4 **Description: Gene expression results.** In the left part of the table, gene expression values were calculated on samples of a single assay (assay 1, 2 or 3). On the right, results are presented per assay but calculations occurred on the entire dataset of 72 samples. For each sample the geometric mean of the biological replicates is presented and (C)NRQ values have been log-transformed. Click here for file

Additional file 5 **Description: Fold differences between 2 CNRQ values of biological replicates.** Samples are grouped according to flower colour (0 = white, 1 = red, 2 = carmine red, 3 = pink). Empty cells indicate one of the biological replicates was discarded after noRT analysis. Click here for file

## References

[B1] De Loose R Flavonoid glycosides in the petals of some *Rhododendron* species and hybrids Phytochem 1968 9 875 879

[B2] De Loose R The flower pigments of the Belgian hybrids of *Rhododendron simsii* and other species and varieties from *Rhododendron* subseries obtusum Phytochem 1969 88 253 259

[B3] Gerats AGM Martin C Stafford HA, Ibrahim RK Flavonoid synthesis in Petunia hybrida: genetics and molecular biology of flower colour Recent advances in phytochemistry 1992 New York: Plenum Press 165 200

[B4] Holton TA Cornish EC Genetics and biochemistry of anthocyanin biosynthesis Plant Cell 1995 7 1071 1083 12242398 10.1105/tpc.7.7.1071 PMC160913

[B5] Mol J Grotewold R Koes RE How genes paint flowers and seeds Trends Plant Sci 1998 3 212 217 10.1016/S1360-1385(98)01242-4

[B6] Winkel-Shirley B Biosynthesis of flavonoids and effects of stress Curr Opin Plant Biol 2002 5 218 223 10.1016/S1369-5266(02)00256-X 11960739

[B7] Schwinn K Venail J Shang Y Mackay S Alm V Butelli E Oyama R Bailey P Davies K Martin C A small family of MYB-regulatory genes controls floral pigmentation intensity and patterning in the genus *Antirrhinum* Plant Cell 2006 18 831 851 10.1105/tpc.105.039255 16531495 PMC1425845

[B8] De Cooman L Everaert ESW Faché P Vande Casteele K Van Sumere CF Flavonoid biosynthesis in petals of *Rhododendron simsii* Phytochem 1993 33 1419 1426 10.1016/0031-9422(93)85102-W

[B9] De Schepper S Debergh P Van Bockstaele E De Loose M Gerats A Depicker A Genetic and epigenetic aspects of somaclonal variation: flower colour bud sports in azalea, a case study South African J Bot 2003 69 117 128

[B10] Heursel J Horn W A hypothesis on the inheritance of flower colours and flavonoids in *Rhododendron simsii* Planch Zeitschrift für Pflanzenzüchtung 1977 79 238 24919349661

[B11] Sasaki N Nishizaki Y Uchida Y Wakamatsu E Umemoto N Momose M Okamura M Yoshida H Yamaguchi M Nakayama M Ozeki Y Itoh Y Identification of the *glutathione S-transferase* gene responsible for flower color intensity in carnations Plant Biotechnol 2012 29 223 227 10.5511/plantbiotechnology.12.0120a

[B12] De Keyser E Lootens P Van Bockstaele E De Riek J Image analysis for QTL mapping of flower colour and leaf characteristics in pot azalea (*Rhododendron simsii* hybrids) Euphytica 2013 189 445 460 10.1007/s10681-012-0809-7

[B13] Nakamura N Fukuchi-Mizutani M Fukui Y Ishiguro K Suzuki K Suzuki H Okazaki K Shibata D Tanaka Y Generation of pink flower varieties from blue *Torenia hybrida* by redirecting the flavonoid biosynthetic pathway from delphinidin to pelargonidin Plant Biotechnol 2010 27 375 383 10.5511/plantbiotechnology.10.0610a

[B14] Boase MR Lewis DH Davies KM Marshall GB Patel D Schwinn KE Deroles SC Isolation and antisense suppression of *flavonoid 3′,5′-hydroxylase* modifies flower pigments and colour in cyclamen BMC Plant Biol 2010 10 107 10.1186/1471-2229-10-107 20540805 PMC3095274

[B15] Nishihara M Nakatsuka T Genetic engineering of flavonoid pigments to modify flower color in floricultural plants Biotechnol Lett 2011 33 433 441 10.1007/s10529-010-0461-z 21053046

[B16] Chen W-H Hsu C-Y Cheng H-Y Chang H Chen H-H Ger M-J Downregulation of putative UDP-glucose: flavonoid 3-*O*-glucosyltransferase gene alters flower coloring in *Phalaenopsis* Plant Cell Rep 2011 30 1007 1017 10.1007/s00299-011-1006-1 21274540

[B17] Kamiishi Y Otani M Takagi H Han D-S Mori S Tatsuzawa F Okuhara H Kobayashi H Nakano M Flower color alteration in the liliaceous ornamental Tricyrtis sp. By RNA interference-mediated suppression of the chalcone synthase gene Mol Breeding 2012 30 671 680 10.1007/s11032-011-9653-z

[B18] Nakatsuka T Mishiba K Kubota A Abe Y Yamamura S Nakamura N Tanaka Y Nishihara M Genetic engineering of novel flower colour by suppression of anthocyanin modification genes in gentian J Plant Physiol 2010 167 231 237 10.1016/j.jplph.2009.08.007 19758726

[B19] Akita Y Kitamura S Hase Y Narumi I Ishizaka H Kondo E Kameari N Nakayama M Tanikawa N Morita Y Tanaka A Isolation and characterization of the fragrant cyclamen *O-methyltransferase* involved in flower coloration Planta 2011 234 1127 1136 10.1007/s00425-011-1466-0 21735197

[B20] Yamamizo C Noda N Ohmiya A Anthocyanin and carotenoid pigmentation in flowers of section *Mina*, subgenus *Quamoclit*, genus *Ipomoea* Euphytica 2012 184 429 440 10.1007/s10681-011-0618-4

[B21] Sui X Gao X Ao M Wang Q Yang D Wang M Fu Y Wang L cDNA cloning and characterization of UDP-glucose: anthocyanidin 3-*O*-glucosyltransferase in *Freesia hybrida* Plant Cell Rep 2011 30 1209 1218 10.1007/s00299-011-1029-7 21318353

[B22] Mizuta D Nakatsuka A Miyajima I Ban T Kobayashi N Pigment composition patterns and expression analysis of flavonoid biosynthesis genes in the petals of evergreen azalea ‘Oomurasaki’ and its red flower sport Plant Breeding 2010 129 558 562

[B23] De Schepper S Debergh P Van Bockstaele E De Loose M Molecular characterisation of flower colour genes in azalea sports (*Rhododendron simsii* hybrids) Acta Hort 2001 552 143 150

[B24] Liu X-J Chuang Y-N Chiou C-Y Chin D-C Shen F-Q Yeh K-W Methylation effect on chalcone synthase gene expression determines anthocyanin pigmentation in floral tissues of two *Oncidium* orchid cultivars Planta 2012 236 401 409 10.1007/s00425-012-1616-z 22391855

[B25] Livak KJ Schmittgen TD Analysis of relative gene expression data using real-time quantitative PCR and the 2(−Delta Delta C(T)) Method Methods 2001 25 402 408 10.1006/meth.2001.1262 11846609

[B26] Bustin SA Benes V Garson JA Hellemans J Huggett J Kubista M Mueller R Nolan T Pfaffl MW Shipley GL Vandesompele J Wittwer CT The MIQE guidelines: *M*inimum *I*nformation for publication of *Q*uantitative real-time PCR *E*xperiments Clin Chemistry 2009 55 611 622 10.1373/clinchem.2008.112797 19246619

[B27] Guttierrez L Mauriat M Guénin S Pelloux J Lefebvre J Louvet R Rusterrucci C Moritz T Guerineau F Bellini C Van Wuytswinkel O The lack of a systematic validation of reference genes: a serious pitfall undervalued in reverse transcription – polymerase chain reaction (RT-PCR) analysis in plants Plant Biotechnol J 2008 6 609 618 10.1111/j.1467-7652.2008.00346.x 18433420

[B28] Guttierrez L Mauriat M Pelloux J Bellini C Van Wuytswinkel O Towards a systematic validation of references in real-time RT-PCR Plant Cell 2008 20 1734 1735 10.1105/tpc.108.059774 18664615 PMC2518241

[B29] Guénin S Mauriat M Pelloux J Van Wuytswinkel O Bellini C Guttierrez L Normalization of qRT-PCR data: the necessity of adopting a systematic, experimental conditions-specific, validation of references J Exp Bot 2009 60 487 493 10.1093/jxb/ern305 19264760

[B30] Vandesompele J De Preter K Pattyn F Poppe B Van Roy N De Paepe A Speleman F Accurate normalization of real-time quantitative RT-PCR data by geometric averaging of multiple internal control genes Genome Biol 2002 3 1 11 10.1186/gb-2002-3-7-research0034 PMC126239 12184808

[B31] Thellin O Elmoualij B Heinen E Zorzi W A decade of improvements in quantification of gene expression and internal standard selection Biotechnol Adv 2009 4 323 333 19472509 10.1016/j.biotechadv.2009.01.010

[B32] Nolan T Hands RE Bustin SA Quantification of mRNA using real-time RT-PCR Nat Protoc 2006 1 1559 1582 10.1038/nprot.2006.236 17406449

[B33] Die JV Roman B RNA quality assessment: a view from plant qPCR studies J Exp Bot 2012 63 6069 6077 10.1093/jxb/ers276 23045609

[B34] Nakatsuka A Mizuta D Kii Y Myajima I Kobayashi N Isolation and expression analysis of flavonoid biosynthesis genes in evergreen azalea Sci Hort 2008 118 314 320 10.1016/j.scienta.2008.06.016

[B35] Baelde HJ Cleton-Jansen AM Van Beerendonck H Namba K High quality RNA isolation from tumors with low cellularity and high extra-cellular matrix component for cDNA microarrays. Application to chondrosarcoma J Clin Pathol 2001 54 778 782 10.1136/jcp.54.10.778 11577126 PMC1731295

[B36] Shultz DJ Craig R Cox-Foster DL Mumma RO Medford JI RNA isolation from recalcitrant plant tissue Plant Mol Biol Rep 1994 12 310 316 10.1007/BF02669273

[B37] Mueller O Hahnenberger K Dittmann M Yee H Dubrow R Nagle R Ilsley D A microfluidic system for high-speed reproducible DNA sizing and quantitation Electrophoresis 2000 21 128 134 10.1002/(SICI)1522-2683(20000101)21:1<128::AID-ELPS128>3.0.CO;2-M 10634479

[B38] Nolan T Hands RE Ogunkolade W Bustin SA SPUD: A quantitative PCR assay for the detection of inhibitors in nucleic acid preparations Anal Biochem 2006 351 308 310 10.1016/j.ab.2006.01.051 16524557

[B39] Vermeulen J De Preter K Lefever S Nuytens J De Vloed F Derveaux S Hellemans J Speleman F Vandesompele J Measurable impact of RNA quality on gene expression results from quantitative PCR Nucl Acids Res 2011 39 1 12 10.1093/nar/gkq742 21317187 PMC3089491

[B40] Schroeder A Mueller O Stocker S Salowsky R Leiber M Gassmann M Lightfoot S Menzel W Granzow M Ragg T The RIN: and RNA integrity number for assigning integrity values to RNA measurements BMC Mol Biol 2006 7 3 10.1186/1471-2199-7-3 16448564 PMC1413964

[B41] Hellemans J Mortier G De Paepe A Speleman F Vandesompele J qBase relative quantification framework and software for management and automated analysis of real-time quantitative PCR data Genome Biol 2007 8 10.1186/gb-2007-8-2-r19 PMC1852402 17291332

[B42] De Keyser E De Riek J Van Bockstaele E Discovery of species-wide EST-derived markers in *Rhododendron* by intron-flanking primer design Mol Breeding 2009 23 171 178 10.1007/s11032-008-9212-4

[B43] Ovstebo R Bente Foss Haug K Lande K Kierulf P PCR-based calibration curves for studies of quantitative gene expression in human monocytes: development and evaluation Clinical Biochem 2003 49 425 432 10.1373/49.3.425 12600954

[B44] Wang QT Xiao W Mindrinos M Davis RW Yeast tRNA as carrier in the isolation of microscale RNA for global amplification and expression profiling Biotechniques 2002 33 788 796 12398187 10.2144/02334st02

[B45] Quattrocchio F Wing JF van der Woude K Souer E de Vetten N Mol J Koes R Molecular analysis of the *anthocyanin2* gene of petunia and its role in the evolution of flower color Plant Cell 1999 11 1433 1444 10449578 10.1105/tpc.11.8.1433 PMC144295

[B46] Streisfeld MA Rausher MD Altered *trans*-regulatory control of gene expression in multiple anthocyanin genes contributes to adaptive flower color evolution in *Mimulus aurantiacus* Mol Biol Evol 2009 26 433 444 10.1093/molbev/msn268 19029190

[B47] Bustin SA Nolan T Pitfalls of quantitative real-time reverse-transcription polymerase chain reaction J Biomol Tech 2004 15 155 166 15331581 PMC2291693

[B48] Fleige S Walf V Huch S Prgomet C Sehm J Pfaffl M Comparison of relative mRNA quantification models and the impact of RNA integrity in quantitative real-time RT-PCR Biotechnol letters 2006 28 1601 1613 10.1007/s10529-006-9127-2 16900335

[B49] D’haene B Hellemans J The importance of quality control during qPCR data analysis Int Drug Disc 2010 18 24

[B50] Imbeaud S Graudens E Boulanger V Barlet X Zaborski P Eveno E Mueller O Schroeder A Auffray C Towards standardization of RNA quality assessment using user-independent classifiers of microcapillary electrophoresis traces Nucl Acids Res 2005 33 e56 10.1093/nar/gni054 15800207 PMC1072807

[B51] Pfaffl MW Fleige S Riedmaier I Validation of lab-on-chip capillary electrophoresis systems for total RNA quality and quantity control Biotechnol Biotechnol Equip 2008 22 839 843

[B52] Denisov V Strong W Walder M Gringich J Wintz H Development and validation of RQI: an RNA quality indicator for the ExperionTM automated gel electrophoresis system Tech Note Bio-Rad 2008 5761

[B53] Krupp G Stringent RNA quality control using the Agilent 2100 bioanalyzer Agilent Technologies application note 2005

[B54] Pico de Coana Y Parody N Fernandez-Caldas E Alonso C A modified protocol for RNA isolation from high polysaccharide containing *Cupressus arizonica* pollen. Applications for RT-PCR and phage display library construction Mol Biotechnol 2009 10.1007/s12033-009-9219-z 19902388

[B55] Philips MA D’auria JC Luck K Gershenzon J Evaluation of candidate reference genes for real-time quantitative PCR of plant samples using purified cDNA as template Plant Mol Biol Rep 2009 27 407 416 10.1007/s11105-008-0072-1 24489433 PMC3906740

[B56] Onate-Sanchez L Vicente-Carbajosa J DNA-free RNA isolation protocols for Arabidopsis thaliana, including seeds and siliques BMC Reseach Notes 2008 1 93 10.1186/1756-0500-1-93 PMC2613888 18937828

[B57] De Santis C Smith-Keune C Jerry DR Normalizing RT-qPCR data: are we getting the right answers? An appraisal of normalization approaches and internal reference genes from a case-study in the Finfish *Lates Calcarifer* Mar Biotechnol 2011 13 170 180 10.1007/s10126-010-9277-z 20309600

[B58] Grunwald U Guo W Fischer K Isayenkov S Ludwig-Muller J Hause B Yan X Kuster H Franken P Overlapping expression patterns and differential transcript levels of phosphate transporter genes in arbuscular mycorrhizal, P_i_-fertilised and phytohormone treated *Medicago truncatula* roots Planta 2009 229 1023 1034 10.1007/s00425-008-0877-z 19169704 PMC2757622

[B59] Laurell H Iacovoni JS Abot A Svec D Maoret J-J Arnal J-F Kubista M Correction of RT-qPCR data for genomic DNA-derived signals with ValidPrime Nucl Acids Res 2012 40 e51 10.1093/nar/gkr1259 22228834 PMC3326333

[B60] Czechowski T Stitt M Altmann T Udvardi MK Scheible WR Genome-wide identification and testing of superior reference genes for transcript normalization in Arabidopsis Plant Physiol 2005 139 5 17 10.1104/pp.105.063743 16166256 PMC1203353

[B61] Popovici V Goldstein DR Antonov J Jaggi R Delorenzi M Wirapati P Selecting control genes for RT-qPCR using public microarray data BMC Bioinforma 2009 10 1186/1471-2105-10-42 10.1186/1471-2105-10-42 PMC2640357 19187545

[B62] Coker JS Davies E Selection of candidate housekeeping controls in tomato plants using EST data Biotechniques 2003 35 740 748 14579739 10.2144/03354st04

[B63] Cruz F Kalaoun S Nobile P Colombo C Almeida J Barros LMG Romano E Grossi-de-Sa MF Vaslin M Alves-Ferreira M Evaluation of coffee reference genes for relative expression studies by quantitative real-time RT-PCR Mol Breeding 2009 23 607 616 10.1007/s11032-009-9259-x

[B64] Klie M Debener T Identification of superior reference genes for data normalisation of expression studies via quantitative PCR in hybrid roses (*Rosa hybrida*) BMC Reserach Notes 2011 4 518 10.1186/1756-0500-4-518 PMC3248381 22123042

[B65] Mallona I Lischewski S Weiss J Hause B Egea-Cortines M Validation of reference genes for quantitative real-time PCR during leaf and flower development in *Petunia hybrida* BMC Plant Biol 2010 10 10.1186/1471-2229-10-4 PMC2827423 20056000

[B66] Regier N Frey B Experimental comparison of relative RT-qPCR quantification approaches for gene expression studies in poplar BMC Mol Biol 2010 11 57 10.1186/1471-2199-11-57 20701777 PMC2930637

[B67] Ramakers C Ruijter JM Lekanne-Deprez RH Moorman AFM Assumption-free analysis of quantitative real-time PCR data Neuroscience Lett 2002 339 62 66 10.1016/s0304-3940(02)01423-4 12618301

[B68] Liu W Saint DA A new quantitative method of real time reverse transcription polymerase chain reaction assay based on simulation of polymerase chain reaction kinetics Anal Biochem 2002 302 52 59 10.1006/abio.2001.5530 11846375

[B69] Liu W Saint DA Validation of a quantitative method for real time PCR kinetics Biochem Biophys Res Comm 2002 294 347 353 10.1016/S0006-291X(02)00478-3 12051718

[B70] Lalam N Estimation of the reaction efficiency in polymerase chain reaction J Theor Biol 2006 242 947 953 10.1016/j.jtbi.2006.06.001 16843498

[B71] Rieu I Powers SJ Real-time quantitative RT-PCR: design, calculations and statistics Plant Cell 2009 21 1031 1033 10.1105/tpc.109.066001 19395682 PMC2685626

[B72] Nordgard O Kvaloy JT Farmen RK Heikkila R Error propagation in relative real-time reverse transcription polymerase chain reaction quantification models: the balance between accuracy and precision Anal Biochem 2006 356 182 193 10.1016/j.ab.2006.06.020 16899212

[B73] Marino JH Cook P Miller KS Accurate and statistically verified quantification of relative mRNA abundances using SYBR Green I and real-time RT-PCR J Immunol Meth 2003 283 291 306 10.1016/S0022-1759(03)00103-0 14659920

[B74] Cook P Fu C Hickey M Han E-S Miller K SAS programs for real-time RT-PCR having multiple independent samples Bioinformatics 2004 37 990 995 10.2144/04376BIN02 15597549

[B75] Derveaux S Vandesompele J Hellemans J How to do successful gene expression analysis using real-time PCR Methods 2010 50 227 230 10.1016/j.ymeth.2009.11.001 19969088

[B76] Kunert R Gach JS Vorauer-Uhl K Engel E Katinger H Validated method for quantification of genetically modified organisms in samples of maize flour J Agricul Food Chem 2006 54 678 681 10.1021/jf052257s 16448167

[B77] Taverniers I Van Bockstaele E De Loose M Cloned plasmid DNA fragments as calibrators for controlling GMOs: diffeent real-time duplex quantitative PCR methods Anal Bioanal Chem 2004 378 1198 1207 10.1007/s00216-003-2372-5 14689155

[B78] Jansen RC Nap JP Genetical genomics: the added value from segregation Trends Genet 2001 17 388 391 10.1016/S0168-9525(01)02310-1 11418218

[B79] Joosen RVL Ligterink W Hilhorst HWM Keurentjes JJB Advances in genetical genomics of plants Curr Genomics 2009 10 540 549 10.2174/138920209789503914 20514216 PMC2817885

[B80] Motulsky H Choosing an appropriate sample size Intuitive Biostatistics 1995 New York: Oxford University Press 195 204

[B81] Shi C Uzarowska A Ouzunova M Landbeck M Wenzel G Lübberstedt T Identification of candidate genes associated with cell wall digestibility and eQTL (expression quantitative trait loci) analysis in a Flint x Flint maize recombinant inbred line population BMC Genomics 2007 8 22 10.1186/1471-2164-8-22 17233901 PMC1785377

[B82] Schwinn KE Davies KM Davies K Flavonoids Plant Pigments and their manipulation Annual Plant Reviews, Volume 14. 2004 Oxford: Blackwell Publishing Ltd 92 149

[B83] Larsen ES Alfenito MR Briggs WR Walbot V A carnation anthocyanin mutant is complemented by the *glutathione S-transferases* encoded by maize *Bz2* and petunia *An9* Plant Cell Rep 2003 21 900 904 12789508 10.1007/s00299-002-0545-x

[B84] De Jong WS Eannetta NT De Jong DM Bodis M Candidate gene analysis of anthocyanin pigmentation loci in the *Solanaceae* Theor Appl Genet 2004 108 423 432 10.1007/s00122-003-1455-1 14523517

[B85] Jung CS Griffiths HM De Jong DM Cheng S Bodis M Kim TS De Jong WS The potato developer (D) locus encodes an R2R3 MYB transcription factor that regulates expression of multiple anthocyanin structural genes in tuber skin Theor Appl Genet 2009 120 45 57 10.1007/s00122-009-1158-3 19779693 PMC2778721

[B86] Kliebenstein DJ West MA van Leeuwen H Loudet O Dioerge RW and St Clair DA: Identification of QTLs controlling gene expression networks defined a priori BMC Bioinforma 2006 7 308 10.1186/1471-2105-7-308 PMC1540440 16780591

[B87] De Keyser E Shu QI Van Bockstaele E De Riek J Multipoint-likelihood maximization mapping on 4 segregating populations to achieve an integrated framework map for QTL analysis in pot azalea (*Rhododendron simsii* hybrids) BMC Mol Biol 2010 11 1 10.1186/1471-2199-11-1 20070894 PMC2837023

[B88] Moore D Dowhan D Manipulation of DNA Current Protocols Mol Biol 2002 59 2.1.1 2.1.10

[B89] Dhanasekaran S Doherty TM Kenneth J Comparison of different standards for real-time PCR-based absolute quantification J Immunol Methods 2010 354 34 39 10.1016/j.jim.2010.01.004 20109462

[B90] Luu-The V Paquet N Calvo E Cumps J Improved real-time RT-PCR method for high-throughput measurements using second derivative calculation and double correction Biotechniques 2005 38 287 293 10.2144/05382RR05 15727135

[B91] Van Ooijen JW MapQTL®5, software for the mapping of quantitative trait loci in experimental populations 2004 Wageningen, The Netherlands: Kyazma BV

[B92] Mantel N The detection of disease clustering and a generalized regression approach Cancer Res 1976 27 209 220 6018555

[B93] Liedloff AC Mantel Nonparametric Test Calculator. Version 2.0 1999 Australia: School of Natural Resource Sciences, Queensland University of Technology

