# Supplementary material for: How to perform RT-qPCR accurately in plant species? A case study on flower colour gene expression in an azalea (Rhododendron simsii hybrids) mapping population
Source: BMC Mol Biol. 2013 Jun 24;14:13. doi: 10.1186/1471-2199-14-13 (PMC3698002; doi:10.1186/1471-2199-14-13)
Supplement: Additional file 1 — RNA concentration and purity. Description: RNA quantity and purity was measured of each biological replicate per sample using a NanoDrop spectrophotometer. For each sample, the assay is indicated in which the sample was analysed. Flower colour is indicated as well (0 = white, 1 = red, 2 = carmine red, 3 = pink). Samples used for analysis in the SPUD-assay and the Experion are indicated with an *. [file 1471-2199-14-13-S1.pdf]

Sample	Assay	Colour	RNA concentration (ng/µl)		A260/280		A260/230	
Replicates			a	b	a	b	a	b
1	2	0	9.23*	69.93	1.85	1.98	2.4	2.22
3	2	1	210.07*	234.54	2.08	1.97	2.18	2.29
6	2	1	5.16*	4.98	1.45	1.52	1.27	1.05
7	2	2	66.31	64.36*	1.97	1.99	2.57	2.45
9	2	2	48.67	77.32	2.06	1.98	2.41	2.23
10	2	0	1.74	2.52	1.43	1.34	0.82	0.82
12	2	1	10.79*	57.79	1.97	2.07	1.74	2.21
13	2	2	1.64	4.62	6.02	2.31	0.80	2.82
14	2	0	12.93	163.94	1.78	1.94	1.81	2.46
16	2	0	179.56*	165.45	2.02	1.99	2.38	2.42
17	2	1	171.12*	145.57	1.98	1.92	2.33	2.52
18	2	1	27.3*	44.92	1.84	1.91	1.94	2.43
19	2	3	26.92	46.27	1.90	1.63	1.85	0.73
21	2	0	5.32	29.61	1.62	1.87	1.70	1.84
22	2	3	1.80	1.86	1.03	0.95	1.28	1.07
24	2	0	36.09	167.01	1.92	1.98	2.57	2.45
25	2	1	9.91	24.03	2.75	1.93	3.02	2.03
26	2	2	10.82	3.69	1.60	1.17	1.45	1.34
28	2	1	83.81	6.00	1.96	2.15	2.33	1.01
29	2	2	10.73	1.86*	1.76	1.31	1.56	0.84
32	2	2	5.38*	9.56	1.31	1.64	0.84	1.13
33	2	0	26.25*	4.21	1.98	1.5	1.76	1.16
36	2	0	7.85	4.81	1.87	1.35	1.73	1.16
38	2	2	15.24*	29.58	2.10	2.01	2.32	2.45
48	3	0	10.04	6.01	2.16	1.92	2.00	1.52
50	3	1	85.25	95.19	2.06	2.02	2.40	2.51
57	3	1	26.18	8.59	1.88	1.97	1.96	2.20
58	3	1	52.53	92.49	2.10	2.16	2.23	2.00
59	3	1	6.17	3.04	1.45	1.90	2.43	2.14
66	2	2	141.04	198.04	1.97	1.96	2.25	2.41
67	2	2	18.46	59.32	1.74	2.08	1.66	2.29
68	3	1	15.24	29.58	2.10	2.01	2.32	2.45
71	3	2	6.83	8.06	1.79	1.53	2.19	1.09
73	3	2	4.86	10.68	1.93	1.64	1.23	2.20
79	3	2	43.84	23.99	1.89	1.85	2.33	2.48
80	3	0	95.41	183.87	1.94	1.99	2.40	2.42
83	2	1	102.22	326.74	1.89	2.04	1.97	2.24
84	3	0	2.34	1.67	1.18	1.52	0.76	0.59
95	3	0	6.44	15.09	1.41	1.79	1.48	1.83
100	3	0	130.47	33.89	1.92	1.87	2.36	2.20
102	1	3	22.43	37.13	2.16	2.14	1.93	2.01
104	3	1	109.97	135.25	1.87	1.88	2.56	2.57
108	3	1	11.73	41.94	2.04	1.96	1.93	2.31
109	3	1	91.62	65.12	1.97	1.94	2.33	2.17

Sample	Assay	Colour	RNA concentration (ng/µl)		A260/280		A260/230	
Replicates			a	b	a	b	a	b
111	3	0	5.83	5.05	1.62	2.13	2.11	2.23
117	3	0	16.65	13.94	1.96	1.83	1.78	1.31
121	1	3	100.32	116.79	2.00	2.08	2.37	2.33
131	1	1	14.31	7.03	2.16	2.28	1.64	1.14
132	1	1	36.61	6.37	1.93	2.94	2.34	1.60
153	1	0	18.55	52.87	2.12	1.93	1.91	2.43
158	1	0	36.76	40.27	2.02	1.89	1.97	2.17
161	1	3	77.25	45.67	1.97	1.96	2.29	1.94
164	1	0	19.39	32.89	1.96	1.92	1.67	1.72
168	2	0	394.47*	30.89	2.01	2.13	2.33	1.87
173	3	2	20.68	18.62	1.67	1.87	2.17	2.51
174	3	0	4.31	10.38	1.51	1.65	2.08	1.94
176	3	0	35.19	71.20	2.01	2.05	2.19	2.17
179	1	0	25.42	9.23	1.87	2.00	2.07	1.40
180	2	1	73.97*	54.17	2.01	1.90	2.36	1.18
185	1	2	150.99	413.35	1.96	1.93	2.44	2.42
190	1	3	128.43	164.60	2.06	1.97	2.21	2.44
191	1	1	8.74	23.93	2.51	2.10	1.63	1.91
197	1	2	32.64	52.24	1.99	1.96	1.88	2.21
201	1	2	83.74	37.29	2.04	2.11	1.87	2.17
209	1	2	41.03	56.71	2.08	2.03	2.12	2.23
213	1	0	124.53	26.00	1.91	1.93	2.42	2.12
220	1	1	18.78	19.16	2.10	1.94	2.08	1.50
234	1	2	246.78	90.88	2.04	1.95	2.14	2.34
236	1	3	575.34	704.98	2.05	2.00	2.33	2.41
250	1	1	30.36	17.58	2.01	2.26	2.28	1.94
98-13-4	1	3	155.57	239.58	1.94	2.03	2.44	2.40
V151	1	3	185.42	221.13	2.04	1.97	2.41	2.42
