# Supplementary figures and images for: How to perform RT-qPCR accurately in plant species? A case study on flower colour gene expression in an azalea (Rhododendron simsii hybrids) mapping population
Source: BMC Mol Biol. 2013 Jun 24;14:13. doi: 10.1186/1471-2199-14-13 (PMC3698002; doi:10.1186/1471-2199-14-13)

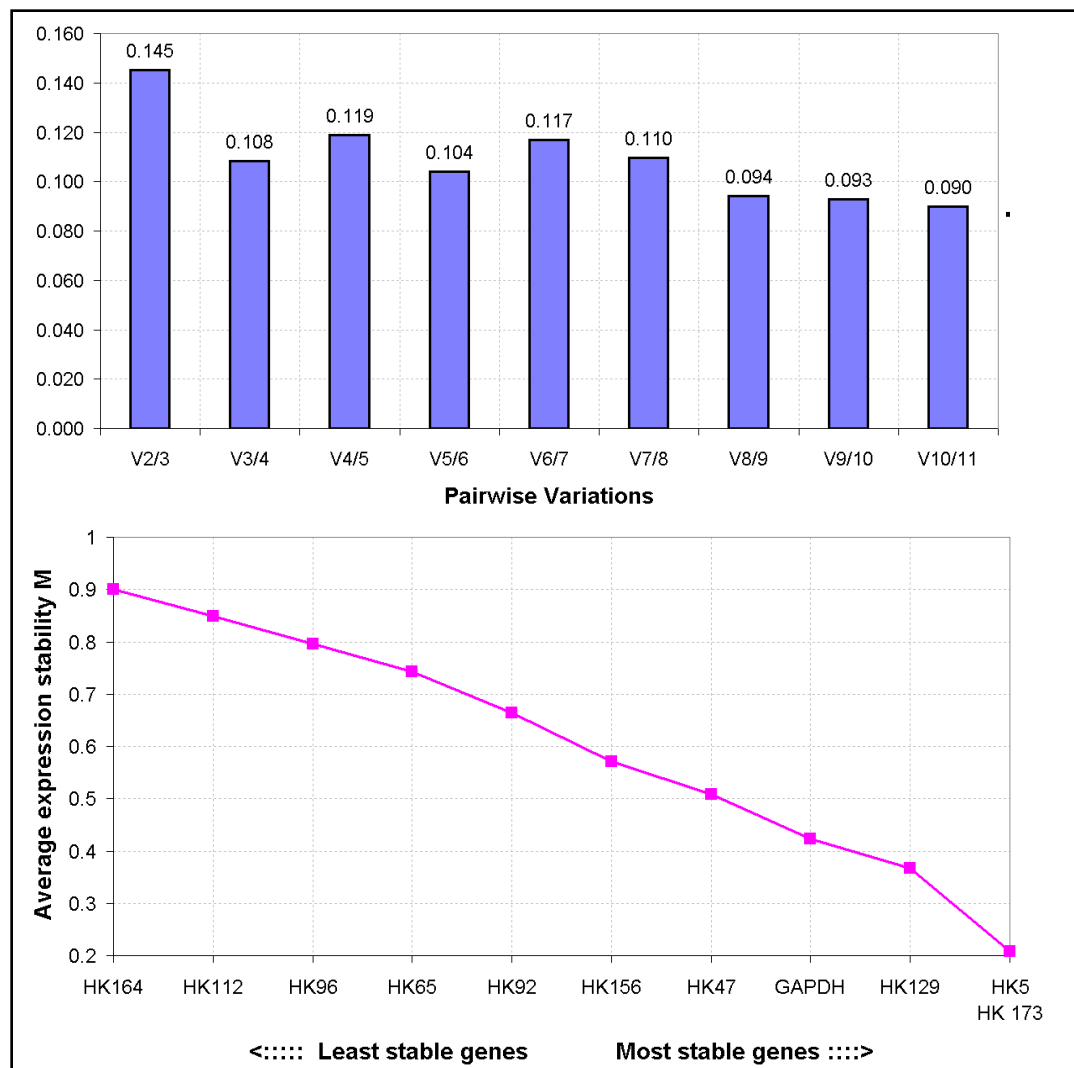

Supplement: Additional file 2 — Evaluation of the optimal number of reference genes for normalization. Description: A cut-off value of 0.15 is proposed (top panel). Average expression stability (M) of the reference genes tested in azalea. M is calculated at each step during stepwise exclusion of the least stable reference gene. Genes are ranked from the least (left) to the most stable (right). Only genes with an M-value < 0.5 are valid in homogeneous samples (lower panel). Both graphs are generated in GeNorm [30]. [file 1471-2199-14-13-S2.pdf]
