# Supplementary material for: How to perform RT-qPCR accurately in plant species? A case study on flower colour gene expression in an azalea (Rhododendron simsii hybrids) mapping population
Source: BMC Mol Biol. 2013 Jun 24;14:13. doi: 10.1186/1471-2199-14-13 (PMC3698002; doi:10.1186/1471-2199-14-13)
Supplement: Additional file 3 — PCR efficiencies of the standard curves. Description: Summary of slopes and derived PCR efficiencies (E) of the standard curves of dilution series analysed on different plates in 3 independent assays. E and the standard deviation on E (SD(E)) were calculated according to the formulas described in Hellemans et al. [41]. [file 1471-2199-14-13-S3.pdf]

Gene	Assay	Slope	E	SD (E)
	1	-3.39	0.97	0.0017
ANS	2	-3.51	0.93	0.0012
	3	-3.52	0.92	0.0027
	1	-3.38	0.97	0.0037
CHS	2	-3.46	0.94	0.0019
	3	-3.46	0.94	0.0010
	1	-4.08	0.76	0.0025
DFR	2	-4.34	0.70	0.0028
	3	-4.34	0.70	0.0028
	1	-3.42	0.96	0.0011
F3H	2	-3.46	0.94	0.0025
	3	-3.47	0.94	0.0020
	1	-3.90	0.81	0.0034
F3'H	2	-3.90	0.81	0.0034
	3	-3.78	0.84	0.0048
	1	-3.37	0.98	0.0022
FLS	2	-3.45	0.95	0.0034
	3	-3.50	0.93	0.0092
	1	-3.43	0.96	0.0021
HK5	2	-3.42	0.96	0.0025
	3	-3.61	0.89	0.0029
	1	-3.48	0.94	0.0019
HK129	2	-3.63	0.89	0.0044
	3	-3.89	0.81	0.0042
