# Supplementary material for: How to perform RT-qPCR accurately in plant species? A case study on flower colour gene expression in an azalea (Rhododendron simsii hybrids) mapping population
Source: BMC Mol Biol. 2013 Jun 24;14:13. doi: 10.1186/1471-2199-14-13 (PMC3698002; doi:10.1186/1471-2199-14-13)
Supplement: Additional file 4 — Description: Gene expression results. In the left part of the table, gene expression values were calculated on samples of a single assay (assay 1, 2 or 3). On the right, results are presented per assay but calculations occurred on the entire dataset of 72 samples. For each sample the geometric mean of the biological replicates is presented and (C)NRQ values have been log-transformed. [file 1471-2199-14-13-S4.pdf]

Sample		Individual analysis (per assay)						Analysis on total dataset (72 samples)					
		ANS	CHS	DFR	F3H	F3’H	FLS	ANS	CHS	DFR	F3H	F3’H	FLS
Assay 1	102	0.451	-0.495	-0.277	-0.221	0.925	-0.193	0.337	-0.209	-0.119	0.021	0.061	-0.083
	121	0.287	-0.420	-0.394	-0.194	0.911	-0.224	0.178	-0.135	-0.206	0.049	0.056	-0.112
	131	0.336	-0.067	0.104	-0.161	0.919	0.022	0.104	0.090	0.188	-0.034	0.028	0.006
	132	0.233	-0.354	-0.327	-0.287	0.689	-0.091	0.206	0.013	-0.010	0.044	-0.004	0.099
	153	0.273	-0.291	-0.338	-0.261	0.961	-0.096	0.082	-0.089	-0.207	-0.095	0.045	-0.068
	158	0.085	-0.046	-0.108	-0.179	1.454	0.143	-0.122	0.131	-0.030	-0.036	0.451	0.148
	161	0.164	-0.092	0.126	-0.150	1.123	0.064	-0.084	0.046	0.081	-0.048	0.098	0.031
	164	0.180	-0.108	0.112	-0.149	1.275	0.068	-0.021	0.078	0.154	0.001	0.304	0.081
	179	0.390	-0.174	-0.032	-0.318	0.909	-0.037	0.222	0.050	0.118	-0.123	0.065	0.013
	185	0.349	-0.274	-0.090	-0.171	1.159	-0.234	0.241	0.011	0.016	0.073	0.247	-0.118
	190	0.149	-0.339	-0.059	-0.363	1.135	0.026	-0.030	-0.127	0.025	-0.186	0.198	0.062
	191	0.363	0.342	0.582	0.245	1.395	0.323	0.207	0.567	0.609	0.434	0.473	0.379

	Sample	Individual analysis (per assay)						Analysis on total dataset (72 samples)					
		ANS	CHS	DFR	F3H	F3’H	FLS	ANS	CHS	DFR	F3H	F3’H	FLS
	197	-0.547	-0.475	-0.422	-0.620	0.959	-0.249	-0.695	-0.243	-0.254	-0.417	0.066	-0.191
	201	0.252	0.155	0.334	-0.001	1.299	0.093	0.105	0.391	0.335	0.197	0.336	0.162
	209	0.393	-0.523	0.107	-0.486	0.540	-0.280	0.298	-0.220	0.234	-0.217	-0.231	-0.152
	213	0.231	-0.116	-0.533	-0.070	0.894	-0.162	0.078	0.119	-0.351	0.125	0.007	-0.096
	220	-0.252	-0.098	-0.189	-0.142	1.270	-0.181	-0.426	0.106	-0.076	0.026	0.316	-0.146
	234	-0.211	-0.476	-0.548	-0.327	0.460	-0.339	-0.408	-0.286	-0.426	-0.177	-0.422	-0.322
	236	-0.270	-0.233	-0.385	-0.198	1.291	-0.212	-0.403	0.014	-0.288	0.008	0.302	-0.133
	250	-0.368	-0.422	-0.291	-0.445	0.927	-0.124	-0.595	-0.267	-0.207	-0.323	-0.025	-0.146
	98-13-4	0.297	-0.690	0.071	-0.007	1.197	-0.084	0.195	-0.392	0.214	0.240	0.333	0.032
	V151	0.725	0.153	0.186	0.285	1.626	0.528	0.532	0.352	0.200	0.439	0.596	0.554
Assay 2	1	0.080	-0.270	0.374	-0.161	0.413	-0.009	-0.098	-0.232	-0.002	-0.199	-0.238	-0.101
	3	0.148	-0.123	-0.166	-0.001	0.424	-0.008	0.008	-0.049	-0.376	-0.007	-0.188	-0.057

Sample	Individual analysis (per assay)						Analysis on total dataset (72 samples)					
	ANS	CHS	DFR	F3H	F3'H	FLS	ANS	CHS	DFR	F3H	F3'H	FLS
6	0.241	0.151	0.568	0.089	0.698	0.269	0.084	0.193	0.288	0.058	0.094	0.186
7	0.211	0.046	0.402	0.262	0.775	-0.034	0.083	0.127	0.102	0.258	0.131	-0.070
9	0.072	-0.164	0.432	-0.127	0.646	-0.055	-0.129	-0.153	0.013	-0.193	-0.066	-0.168
10	0.375	0.422	0.537	0.176	0.820	0.354	0.157	0.392	0.302	0.081	0.191	0.204
12	0.214	-0.076	0.097	0.057	0.505	0.176	0.065	-0.010	-0.159	0.041	-0.113	0.112
13	0.254	-0.067	0.192	0.093	0.589	0.005	0.135	0.022	0.051	0.099	0.045	-0.032
14	0.326	-0.074	0.644	-0.019	0.434	0.097	0.154	-0.025	0.223	-0.047	-0.201	0.020
16	0.291	0.001	0.071	0.290	0.423	0.185	0.138	0.064	-0.206	0.266	-0.198	0.122
17	0.180	-0.027	0.021	0.166	0.994	0.332	0.017	0.023	-0.267	0.132	0.268	0.249
18	0.244	-0.373	0.231	-0.317	0.150	-0.106	0.013	-0.374	-0.154	-0.396	-0.505	-0.238
19	0.209	-0.145	-0.302	0.061	0.117	-0.145	0.093	-0.044	-0.406	0.078	-0.416	-0.164
21	-0.154	0.041	0.329	-0.135	0.833	0.049	-0.317	0.065	0.012	-0.179	0.145	-0.048

Sample	Individual analysis (per assay)						Analysis on total dataset (72 samples)					
	ANS	CHS	DFR	F3H	F3'H	FLS	ANS	CHS	DFR	F3H	F3'H	FLS
22	0.305	0.178	0.634	0.180	0.248	0.176	0.176	0.245	0.440	0.171	-0.220	0.121
24	-0.194	-0.769	0.083	-0.424	0.098	-0.289	-0.366	-0.720	-0.242	-0.464	-0.519	-0.380
25	-0.328	0.011	-0.149	-0.006	0.850	-0.150	-0.453	0.064	-0.321	-0.028	0.190	-0.212
26	0.204	-0.108	0.328	-0.095	0.276	0.155	-0.016	-0.119	0.023	-0.182	-0.339	0.013
28	0.124	-0.089	0.629	-0.053	0.349	0.150	-0.091	-0.098	0.223	-0.139	-0.293	0.011
29	-0.249	-0.112	0.111	0.103	0.101	-0.234	-0.273	0.050	0.000	0.180	-0.332	-0.189
32	0.229	-0.034	-0.268	0.329	0.839	0.195	0.093	0.042	-0.409	0.314	0.192	0.142
33	-0.018	0.023	0.321	0.068	0.513	0.024	-0.157	0.077	0.064	0.045	-0.081	-0.043
36	0.000	-0.070	0.528	-0.165	0.502	-0.075	-0.138	-0.013	0.252	-0.179	-0.074	-0.139
38	0.282	-0.270	0.134	-0.087	-0.072	-0.203	0.149	-0.177	-0.113	-0.077	-0.585	-0.233
66	0.432	0.645	0.940	0.638	1.406	0.583	0.261	0.672	0.456	0.589	0.631	0.495
67	0.223	0.103	0.870	-0.034	0.609	0.267	-0.003	0.086	0.336	-0.122	-0.113	0.125

	Sample	Individual analysis (per assay)						Analysis on total dataset (72 samples)					
		ANS	CHS	DFR	F3H	F3'H	FLS	ANS	CHS	DFR	F3H	F3'H	FLS
	83	0.519	0.475	1.169	0.534	1.049	0.597	0.345	0.512	0.627	0.492	0.326	0.512
	168	0.168	0.024	0.120	-0.035	0.630	0.253	-0.042	0.024	-0.239	-0.108	-0.090	0.126
	180	0.202	-0.803	0.713	-0.555	-0.516	-0.381	0.113	-0.647	0.333	-0.485	-0.942	-0.363
Assay 3	48	-0.384	0.040	-0.064	0.230	0.277	-0.282	-0.304	0.168	-0.205	0.121	0.184	-0.043
	50	0.048	0.049	0.397	0.333	0.118	0.187	0.119	0.200	0.077	0.242	0.030	0.423
	57	-0.356	-0.724	0.556	-0.440	-1.301	-0.603	-0.286	-0.561	0.129	-0.518	-1.188	-0.350
	58	0.027	-0.241	-0.303	0.192	-0.459	-0.112	0.121	-0.054	-0.462	0.130	-0.466	0.161
	59	0.273	0.319	0.420	0.362	0.154	-0.040	0.257	0.375	0.130	0.187	0.026	0.129
	68	0.362	0.036	0.284	0.349	-0.340	-0.008	0.344	0.114	-0.049	0.184	-0.433	0.166
	71	0.003	0.239	0.138	0.462	0.379	-0.361	0.009	0.305	-0.084	0.290	0.230	-0.170
	73	-0.087	-0.412	0.060	-0.149	-0.520	-0.412	-0.075	-0.310	-0.217	-0.288	-0.587	-0.214
	79	-0.116	-0.400	0.034	0.104	-0.762	-0.448	0.000	-0.193	-0.191	0.060	-0.717	-0.144

Sample	Individual analysis (per assay)						Analysis on total dataset (72 samples)					
	**ANS**	**CHS**	**DFR**	**F3H**	**F3'H**	**FLS**	**ANS**	**CHS**	**DFR**	**F3H**	**F3'H**	**FLS**
80	-0.008	-0.009	0.100	0.447	0.089	-0.239	0.064	0.143	-0.151	0.349	0.003	0.016
84	-0.027	-0.095	0.014	-0.033	-0.106	-0.051	-0.177	-0.179	-0.259	-0.348	-0.327	-0.036
95	0.046	-0.214	0.288	-0.153	-0.210	-0.240	0.025	-0.145	-0.078	-0.316	-0.343	-0.076
100	0.261	-0.082	0.139	0.312	-0.063	-0.273	0.296	0.054	-0.173	0.201	-0.164	-0.036
104	-0.281	-0.309	-0.217	0.093	-0.481	-0.141	-0.089	-0.039	-0.306	0.116	-0.400	0.214
108	-0.120	-0.211	0.578	-0.168	-0.236	-0.168	-0.127	-0.136	0.112	-0.324	-0.375	-0.002
109	-0.168	0.145	0.269	0.372	0.366	-0.370	-0.137	0.239	-0.064	0.226	0.198	-0.160
111	0.282	0.443	0.263	0.470	0.178	0.255	0.219	0.447	-0.040	0.244	-0.001	0.362
117	-0.032	-0.307	0.388	0.141	-0.232	-0.258	0.057	-0.128	0.078	0.072	-0.267	0.014
173	-0.293	-0.554	0.667	-0.499	-0.784	-0.541	-0.198	-0.368	0.227	-0.545	-0.781	-0.262
174	0.104	-0.055	0.344	0.253	-0.251	-0.169	0.121	0.047	0.017	0.112	-0.335	0.033
176	-0.246	-0.060	0.307	-0.044	-0.061	-0.201	-0.239	0.021	-0.131	-0.195	-0.230	-0.025
