# Supplementary material for: How to perform RT-qPCR accurately in plant species? A case study on flower colour gene expression in an azalea (Rhododendron simsii hybrids) mapping population
Source: BMC Mol Biol. 2013 Jun 24;14:13. doi: 10.1186/1471-2199-14-13 (PMC3698002; doi:10.1186/1471-2199-14-13)
Supplement: Additional file 5 — Description: Fold differences between 2 CNRQ values of biological replicates. Samples are grouped according to flower colour (0 = white, 1 = red, 2 = carmine red, 3 = pink). Empty cells indicate one of the biological replicates was discarded after noRT analysis. [file 1471-2199-14-13-S5.pdf]

Colour	Sample	*ANS*	*CHS*	*DFR*	*F3H*	*F3’H*	*FLS*
0	1	1.02	1.10	3.55	1.01	3.21	1.01
0	10	1.95	1.98	2.04	2.03	1.81	1.87
0	14	2.49	1.60	7.79	1.06	1.59	1.15
0	16	1.05	1.05	1.17	1.14	1.11	1.01
0	21	1.40	1.70	1.30	1.58	1.65	1.57
0	24	1.19	6.24	3.12	3.70	6.20	2.52
0	33	1.24	1.12	1.64	1.14	1.27	1.04
0	36	6.39	4.90	6.06	3.22	1.83	4.86
0	48	1.29	1.13	1.10	1.03	1.00	1.00
0	80	1.02	1.08	1.04	1.12	1.17	1.25
0	84	1.05	1.73	1.02	2.27	1.64	1.17
0	95	1.26	1.18	1.17	1.08	1.18	1.04
0	100	1.14	1.26	1.07	1.67	1.21	1.16
0	111	1.07	1.07	1.70	1.45	1.55	1.28
0	117	1.05	1.17	1.77	1.23	1.56	1.02
0	153	1.07	1.07	1.04	1.02	1.13	1.04
0	158	1.32	1.18	1.26	1.24	1.56	1.11
0	164	1.41	1.60	1.23	1.62	1.41	1.25
0	168	1.01	1.13	3.12	1.10	2.86	1.01
0	174	1.25	1.01	1.53	1.43	1.09	1.01
0	176	2.06	2.31	1.43	3.66	3.67	1.49
0	179	1.42	1.18	1.22	1.07	1.07	1.22
0	213	1.29	1.07	1.45	1.38	1.42	1.06
1	3	1.02	1.17	1.21	1.00	1.29	1.01
1	6	1.20	1.62		1.24	1.17	1.42
1	12	1.18	1.48	1.69	1.42	1.80	1.18
1	17	1.09	1.17	1.61	1.25	1.20	1.13
1	18	1.62	1.07	1.13	1.12	1.10	1.04
1	25	2.46	1.11	1.39	1.48	1.11	1.29
1	28	1.11	1.86	1.29	2.40	1.09	2.03
1	50	1.25	1.25	2.07	1.13	1.31	1.33
1	57	3.61	2.69	3.04	3.23		2.24
1	58	1.04	1.27	1.15	1.27	1.13	1.16
1	59	2.50	1.68	2.51	1.25	1.67	1.78
1	68	1.01	1.15	1.54	1.13	1.66	1.22
1	83	1.36	1.49	1.07	1.63	3.36	1.42
1	104	1.18	1.03	2.38	1.06	1.39	1.02
1	108	1.86	1.53	1.67	1.28	1.11	1.44
1	109	1.61	1.03	1.11	1.59	1.30	1.47
1	131	1.11	1.99	1.13	2.70	10.12	1.43
1	132	1.39	1.16	1.07	1.03	1.82	1.28
1	180	1.26	1.12	1.14	1.08	2.50	1.40
1	191	1.44	1.25	1.30	1.37	1.23	1.34
1	220	1.08	1.13	1.06	1.31	1.06	1.01
1	250	2.79	1.67	2.10	2.03	1.59	1.46

Colour	Sample	*ANS*	*CHS*	*DFR*	*F3H*	*F3'H*	*FLS*
2	7	1.03	1.17		1.19	1.10	1.12
2	9	1.75	1.83	1.10	1.88	3.52	1.20
2	13	1.71	1.43	1.56	1.32	1.96	1.08
2	22	1.83	1.12	3.65	1.43	8.03	1.37
2	26	1.35	1.94	2.07	1.83	2.35	1.34
2	29	1.48	1.22	1.76	1.29	1.51	1.33
2	32	1.16	1.16	1.38	1.32	2.12	1.12
2	38	1.02	1.04	1.16	1.05	1.22	1.13
2	66	1.77	1.39	1.79	1.25	1.05	1.46
2	67	1.07	1.41	1.29	1.35	1.98	1.13
2	71	1.10	1.00	1.03	1.19	1.07	1.01
2	73	1.45	1.60	1.56	1.99	2.44	2.22
2	79	1.39	1.12	2.07	1.17	1.82	1.40
2	173	1.59	1.33	1.38	1.17	1.27	1.07
2	185	1.54	1.05	1.08	1.22	1.28	1.33
2	197	1.62	1.12	1.31	1.17	1.41	1.13
2	201	1.11	1.11	1.37	1.18	1.09	1.37
2	209	1.21	2.51	2.83	1.96	1.86	1.09
2	234	11.63	7.87	3.77	8.07	14.11	3.25
3	19	1.23	1.10		1.16	1.17	1.14
3	102	1.20	1.58	1.38	1.31	1.36	1.12
3	121	2.85	1.12	1.08	1.06	1.83	1.44
3	161	1.18	1.24	1.30	1.09	1.48	1.35
3	190	1.06	1.57	1.07	2.41	2.25	1.35
3	236	1.57	1.37	1.14	1.30	1.13	1.37
3	98-13-4	1.18	1.13	1.06	1.01	1.01	1.05
3	V151	1.41	1.02	1.53	1.06	1.39	1.01
	**Mean**	1.64	1.56	1.76	1.58	2.04	1.36
	**Median**	1.26	1.18	1.38	1.26	1.41	1.22
	**Min**	1.01	1.00	1.02	1.00	1.00	1.00
	**Max**	11.63	7.87	7.79	8.07	14.11	4.86
